# Sex differences in muscle activity and motor variability in response to a non-fatiguing repetitive screwing task

**DOI:** 10.1186/s13293-020-0282-2

**Published:** 2020-01-28

**Authors:** Tessy Luger, Robert Seibt, Monika A. Rieger, Benjamin Steinhilber

**Affiliations:** Institute of Occupational and Social Medicine and Health Services Research, University Hospital Tübingen, University of Tübingen, Wilhelmstraße 27, DE-72074 Tübingen, Germany

**Keywords:** Manual material handling, Electromyography, Motor learning, Motor control, Motor variability, Sex differences, Cycle-to-cycle variability, Upper limb, Adaptation

## Abstract

**Background:**

Musculoskeletal disorders are more prevalent among women than among men, which may be explained by aspects of motor control, including neuromuscular requirements and motor variability. Using an exploratory approach, this study aimed to evaluate sex differences in neuromuscular responses and motor variability during a repetitive task performed on 3 days.

**Methods:**

Thirty women and 27 men performed the non-fatiguing, repetitive, 1-h screwing task. For neuromuscular responses, the mean and difference values of static, median, and peak percentile muscle activity levels (normalized to a reference voluntary contraction force) and, for motor variability, the mean and difference values of relative and absolute cycle-to-cycle variability across days were compared between both sexes for each muscle. A mixed-design analysis of variance was used to assess differences between both sexes.

**Results:**

The non-fatiguing character of the screwing task was confirmed by the absence of decreased force levels in maximal voluntary contractions performed before and after the task and by absence of electromyographic signs of muscle fatigue. The static and median muscle activity levels tended to be higher among women (on average 7.86 and 27.23 %RVE) than men (on average 6.04 and 26.66 %RVE). Relative motor variability of the flexor and biceps muscles and absolute motor variability of both upper arm muscles were lower in women (on average 0.79 and 29.70 %RVE) than in men (on average 0.89 and 37.55 %RVE). The median activity level of both upper arms muscles tended to decrease within days among women (on average - 2.63 %RVE) but increase among men (on average + 1.19 %RVE). Absolute motor variability decreased within days among women (on average - 5.32 to - 0.34%RVE), whereas it tended to decrease less or increase within days among men (on average - 1.21 to + 0.25 %RVE).

**Conclusion:**

Women showed higher levels of muscle activity and lower initial relative and absolute motor variability than males when performing the same occupational task, implying women may have a higher risk for developing disorders and point to both sexes using different intrinsic motor control strategies in task performance. Clearly, biological aspects alone cannot explain why women would be at higher risk for developing disorders than men. Therefore, a wider range of individual and environmental factors should be taken into account for optimizing work station designs and organizations by taking into account sex differences.

## Introduction

Musculoskeletal disorders (MSD) are reported to be more prevalent among the female than among the male working population [1, 2]. Several factors may play a role in the higher prevalence of MSD among women than among men, including not only biological factors [3], but also societal, organizational, and individual factors [4]. A complex interaction of these factors may result in men and women having different workloads while performing the same task and having different neuromuscular responses while having the same workloads [4]. In the field of workplace design, for some occupational tasks, a differentiation between the two sexes has already been established, because it is well known that males and females differ in their functional characteristics (e.g., muscle strength) [5, 6] and anthropometrics (e.g., body height) [5]. However, checklists that assess work-related risk in repetitive work do not distinguish between men and women. Examples of the most common checklists are the Hand Activity Level Threshold Limit Values (HAL TLV) [7, 8], the Key Indicator Method for Manual Handling Operations (KIM-HMO) [9, 10], and the revised Occupational Repetitive Action Checklist method (OCRA) [11].

Motor control could play an important role in the risk for developing MSD, as males and females may adopt different motor strategies when performing the same dynamic task [12]. Motor control in women and men has recently received increased attention to better understand sex differences related to MSD. Two fundamental properties of motor control refer to (1) the ability to perform and accomplish a movement, which can be evaluated by biomechanical and neuromuscular responses to (work) tasks, and to (2) the variability that characterizes the details of movement execution [13–15], which actually is an inherent feature of an individual motor control system [16].

With respect to neuromuscular responses to work tasks, women showed a higher upper trapezius muscle activity during a 34-min box folding task than men [12]. In repetitive industrial tasks, women had higher forearm extensor peak muscle activity (39 %MVE) than males (27 %MVE) [17]. The same group of forearm muscles had a higher activity among women when performing 5-min computer tasks [18] and when performing house painting [19]. All four studies normalized their muscle activity to a maximal voluntary contraction, indicating that all these findings are related to the muscle strength of both sexes, which is shown to be lower in females than in males [19, 20].

With respect to the size of motor variability, cycle-to-cycle parameters of muscle activity, movement, and force are often used. When measuring force output during repetitive isometric elbow flexions, women showed lower motor variability than their male counterparts [21]. In a 6-to-7-min repetitive pointing task, the cycle-to-cycle coefficient of variation of the biceps brachii muscle activity was lower in females than in males [22]. Within the same pointing task, both males and females had similar baseline trapezius muscle activation variability but males increased their variability more than females [22]. These differences in motor variability between males and females may reflect a different adaptation of motor control strategies in performing repetitive tasks [21, 22].

In a previous paper, we have demonstrated that individuals performing a repetitive task on 3 different days showed decreasing levels of muscle activity across the 3 days [23]. This result may imply that motor learning influenced the strategies with which the individuals performed the repetitive screwing task, which was also concluded by Moreno-Briseño et al. [24]. Using an exploratory approach, we performed a secondary analysis of the dataset from our previous publication [23] to now identify sex differences with respect to motor control. The aim of the current study was to evaluate sex differences in the neuromuscular response and motor variability during a 1-h repetitive screwing task. We focused on the levels and changes of muscle activity (10^th^, 50^th^, and 90^th^ percentiles) and the levels and changes of the size of motor variability (cycle-to-cycle standard deviation and coefficient of variation) during the repetitive task performed on 3 days. Based on previous studies, we hypothesized that females (1) on average would have higher muscle activity levels and lower motor variability on the 3 days compared to males, and (2) would show a different adaptation pattern by less clear changes in muscle activity and motor variability than males within each of the 3 days.

## Methods

### Participants

Originally, 65 subjects were recruited, but 8 dropped out due to methodological or organizational issues. The final study sample counted 57 healthy subjects (30 F and 27 M) without acute or cardiovascular diseases, impaired range of motion of the neck and upper extremities, or neurological impairments. The anthropometrics of male and female participants are displayed in Table 1.

**Table 1:** Results of the Mann-Whitney test on the anthropometric data of male and female participants with corresponding effect size *r* (Pearson’s correlation coefficient)

	All (*n* = 57)	Men (*n* = 27)	Women (*n* = 30)	Mann-Whitney test		
				*U* value	*p* value	*r*
Age [years]	28.0 ± 24.5	27.0 ± 16.0	28.0 ± 29.3	436.0	0.620	0.07
Body height [cm]	175.0 ± 14.5	181.0 ± 10.0	167.0 ± 8.0	13.0	0.000*	− 0.83‡
Body weight [kg]	73.5 ± 19.0	79.5 ± 13.0	63.0 ± 16.4	131.0	0.000*	− 0.58‡
BMI [kg·m^-2^]	23.1 ± 4.5	24.1 ± 4.1	22.7 ± 5.2	341.0	0.306	− 0.14
Handedness	53 right; 4 left	23 right; 3 left	30 right; 1 left	–	–	–
Sport [hours/week]	4.0 ± 4.8	5.0 ± 8.3	4.0 ± 2.6	286.0	0.085	− 0.23

*Significant *p* value, *α* = 0.05. ^‡^Large effect size, *r* ≥ 0.5. *n* number of subjects in statistical model

### Experimental protocol

For 1 h, participants performed a repetitive screwing task involving grasping and forearm rotation, in which several hand-arm muscles, including the Mm. triceps brachii, biceps brachii, extensor digitorum, and flexor carpi radialis, are involved [25, 26]. The contribution of each muscle to the screwing task is different; the M. triceps brachii is responsible for providing the forward directed force, the M. biceps brachii for supinating the arm and lifting the forearm, the M. extensor digitorum for stabilizing the wrist and providing grip support, and the M. flexor carpi radialis for gripping the devices and supporting wrist supination [27]. The height of the objects handled was adjusted to the participant’s elbow-height when standing in an upright posture. The experimental task consisted of screwing and fastening 6 screws into 12 vertical rows (see [23] for a picture of the task set-up). The 12 vertical rows represent 12 work cycles, which lasted 270 s each, representing a pace of MTM-85 according to the standardized, predetermined motion-time measurement system (MTM) [28]. This work pace, which was the same for all subjects, was visualized as a vertical bar on a screen in front of the subject, showing the time left to fulfill each work cycle. The instructions to the subject were to perform the task according to the predetermined MTM-85 work pace, without being too fast. One work cycle consisted of (1) screwing in 6 screws on a wooden plate using a T-handle screwdriver (e.g., T-handle 336, T15, handle cross size 80 mm, shaft length 200 mm, 162 g incl. 3-g bit, WiHa, Germany), (2) pressing a buzzer, (3) fastening the 6 screws using a torque screw driver (7443 pistol, 232 g incl. 3-g bit, 5 Nm, Wera, Germany), and (4) pressing the buzzer again.

Participants performed the task with the dominant hand on three separate days with 2 to 7 days in between. The first day was preceded with a 10-min familiarization period. Before task initiation, participants were prepared for the measurements, i.e., their skin was cleaned and the electrodes were attached and they performed reference contractions necessary for electromyographic recordings. Before and directly after the screwing task, maximum voluntary contractions of the extensor digitorum and flexor carpi radialis muscles were performed.

### Data acquisition and data analysis

#### Electromyography

After shaving the skin and preparing it with an abrasive paste (Skin Prep Gel, Nuprep®, Aurora, USA), surface electrodes (Ag/AgCl, 35 × 26 mm, 15-mm active area diameter, Kendall^TM^ H93SG ECG Electrodes, Covidien, Zaltbommel, the Netherlands) were placed in a bipolar configuration (inter-electrode center-to-center distance 26 mm) on the dominant biceps brachii (BIC), triceps brachii (TRI), extensor digitorum (EXT), and flexor carpi radialis (FLEX). A ground electrode was placed on the seventh cervical vertebra. Electromyographic (EMG) data were collected using a data analyzer with data logger (PS11-UD, THUMEDI® GmbH & Co. KG, Thum-Jahnsbach, Germany; CMMR > 96 dB; overall effective sum of noise < 0.9 μV RMS). The EMG signals were differential amplified, analog filtered (high-pass filter, 4^th^ order, − 3 dB at 4 Hz; low-pass filter, 11^th^ order, − 3 dB at 1300 Hz), and sampled (4096 Hz). Synchronous to data storage, EMG signals were real-time transformed into the frequency domain (1024-point Fast Fourier Transformation, Bartlett-window, 50% overlap), digitally high-pass filtered (11^th^ order, 20 Hz), and digitally average-filtered to remove power line interference (11^th^ order, 50 Hz and its first seven harmonics) by replacing it by the spectral values of a 4-Hz wide band around its center frequency by means of both spectral neighbors. The median power frequency (MPF [Hz]) and the root-mean-square (RMS [μV]) were real-time calculated from the power spectrum and stored synchronously to the raw data by the PS11 device.

Participants performed submaximal reference voluntary contractions (RVC) with fixed force levels for EMG normalization, during which the study leader was verbally encouraging the subject to keep the set force level to the best of their capacity for 15 s. While seated upright in a custom-developed device with the upper arm along the upper body and the forearm placed horizontally, participants performed 4 RVCs. The participants were instructed to resist against set force levels determined by a force cell positioned underneath a cushion below the distal end of their forearm for the BIC and TRI or below the hand for the EXT and FLEX. The force level was displayed on a monitor that was connected to the force cell to provide the participant visual feedback. Participants flexed their elbow against 110-N resistance and extended their elbow against 80-N resistance for the BIC and TRI reference contractions, respectively. Wrist extension and flexion reference contractions were performed against a 60-N resistance for both the EXT and FLEX. Participants had a rest period of ~ 1 min in between subsequent RVCs. During the contractions, RMS was recorded and the middle 10 s of a steady-state period was averaged and used for EMG normalization, expressed as a percentage (% RVE) [29].

#### Maximum voluntary contraction

Prior to the screwing task, participants performed 5-s maximal voluntary contractions (MVCs) to assess maximal force levels of the four muscles before the experimental task. For the forearm muscles, i.e., EXT and FLEX, the maximal force level was also determined after the experimental task. To assess the maximal force levels, the task set-up of the RVCs was used, as described in the previous section. The study leader verbally encouraged the subject to perform maximally.

#### Muscle activity

From the normalized EA, the static, median, and peak levels of muscle activity were calculated as the 10^th^ percentile (RMS_10_), 50^th^ percentile (RMS_50_), and 90^th^ (RMS_90_) percentile, respectively. These 3 parameters were calculated for the screwing of rows 2, 3, 11, and 12 (i.e., 1 row representing 1 work cycle). The average of rows 2 and 3 reflected the start of the repetitive task, whereas the average of rows 11 and 12 reflected the end of the repetitive task.

The absolute and relative cycle-to-cycle variability, reflecting the size of motor variability, were calculated for the not-normalized RMS. Absolute variability as the pooled cycle-to-cycle standard deviation (RMS_SD_) and relative variability as the pooled cycle-to-cycle standard deviation divided by the mean (coefficient of variation; RMS_CV_) [30]: the square root of the average variance over the 6 screws for rows 2 and 3 and of rows 11 and 12 was calculated and divided by their mean as start and end, respectively.

For each of the five parameters (RMS_10_, RMS_50_, RMS_90_, RMS_SD_, RMS_CV_), the mean over rows 2 to 12 was calculated as summary statistic per day. For each parameter, the difference between start and end and the mean values of the 1-h experimental task were used for further statistical analysis.

### Fatigue

For determining manifestations of muscle fatigue, electromyographic and force data were analyzed. In case of the electromyographic signals, the difference between the start (average of rows 2 and 3) and end values (average of rows 11 and 12) of RMS_50_ and MPF during screwing were calculated. An increased RMS_50_ concomitant with a decreased MPF within the 1-h screwing task would indicate that this muscle developed signs of fatigue [31]. The MPF of the triceps brachii was excluded due to its too low quality resulting from the generally extremely low EMG recordings of <20 μV. The difference values of RMS_50_ and MPF for EXT, FLEX, and BIC were used for further statistical analysis.

In case of the force signals, the amount of force was determined by calculating the force levels of the MVCs of the extensor digitorum and flexor carpi radialis muscles before and after the experimental task. The difference between before and after the experimental task was used for further statistical analysis.

### Statistical analysis

We checked the normal distribution of the RMS, MPF, and force values by inspecting the histograms, skewness, and kurtosis values, and standardized Shapiro-Wilk tests [32, 33]. Since most of the RMS and force values were positively skewed, we transformed these data using the natural logarithm (ln). The MPF values were normally distributed and therefore not transformed.

### Fatigue

Force decrease as sign for fatigue of EXT and FLEX was statistically evaluated by testing the non-transformed change in force within days against zero. Manifestation of muscle fatigue of EXT, FLEX, and BIC was statistically evaluated by testing the non-transformed change in RMS_50_ and MPF within days against zero. The evaluations were carried out using the non-parametric One-Sample Wilcoxon Signed Rank Test, for which the data were stratified by sex and day.

### Force differences between men and women

To check for an association between sex and maximal force, we statistically tested whether the non-transformed maximal force level during the MVCs performed before the experimental task were different between the two sexes. For each muscle, we carried out a non-parametric independent-samples analysis (i.e., Mann-Whitney *U* test), for which the data were stratified by day.

### Effect of sex on muscle activity and motor variability

We used a mixed-design analysis of variance (mixed ANOVA) model to detect differences in the transformed start, difference and mean values of muscle activity (RMS_10_, RMS_50_, RMS_90_), and motor variability (RMS_SD_, RMS_CV_) across days (within-subject factor), between males and females (sex as between-subject factor). In this model, subject was assigned as a random factor and the ln-transformed dependent variables were used.

We used SPSS (IBM SPSS Statistics 25.0) to perform the analyses and set the level of significance at *p* < 0.05.

## Results

Due to failed or unreliable recordings, data of a different number of participants was available for each parameter, which is mentioned in Additional file 1. All graphs visualizing the results were created using the original, non-transformed data.

### Signs of fatigue

#### Force decrease as sign for fatigue

Among men, the EXT showed a significant increase in force within day 3, pointing to no fatigue. Both the EXT and FLEX showed no signs of fatigue based on decreased force levels, as tested with one-sample Wilcoxon signed rank tests (Table 2).

**Table 2:** Results of the one-sample Wilcoxon signed-rank test for signs of fatigue with corresponding effect size *r* (Pearson’s correlation coefficient)

Muscle	Group	Outcome	Day 1						Day 2						Day 3					
			*N*	Median (IQR)		Wilcoxon singed-rank test				Median (IQR)		Wilcoxon singed-rank test				Median (IQR)		Wilcoxon singed-rank test		
				Pre	Post	*W* value	*p* value	*r*	*n*	Pre	Post	*W* value	*p* value	*r*	*n*	Pre	Post	*W* value	*p* value	*r*
Force																				
Extensor	Men	Force [N]	27	180.17 (57.33)	169.17 (69.00)	190.0	0.981	0.00	27	183.00 (54.00)	178.33 (56.33)	165.0	0.564	− 0.11	27	172.67 (56.50)	175.83 (70.33)	253.5	0.048*	0.38
	Women	Force [N]	30	102.25 (35.50)	94.50 (30.75)	180.5	0.285	− 0.20	30	105.75 (27.42)	105.58 (30.88)	186.0	0.339	− 0.17	30	105.75 (23.04)	104.92 (25.58)	265.5	0.497	0.12
Flexor	Men	Force [N]	27	210.50 (100.33)	196.83 (85.00)	136.5	0.207	− 0.24	27	218.67 (100.83)	219.00 (99.17)	184.5	0.914	− 0.02	27	231.83 (116.50)	222.92 (86.21)	189.5	0.722	0.07
	Women	Force [N]	30	108.08 (35.71)	111.58 (34.79)	239.0	0.894	0.02	30	124.25 (41.75)	126.42 (40.62)	167.5	0.181	− 0.24	30	127.92 (50.83)	132.83 (45.21)	240.0	0.877	0.03
Electromyography: amplitude and frequency																				
Extensor	Men	RMS_50_ [%RVE]	27	49.66 (38.10)	49.37 (38.35)	151.0	0.361	− 0.18	27	49.87 (31.64)	44.78 (31.06)	87.0	0.042*	− 0.39†	27	43.36 (33.20)	46.65 (30.55)	150.0	0.349	− 0.18
		MPF [Hz]	27	99.00 (18.50)	98.50 (13.50)	151.0	0.533	− 0.12	27	100.00 (13.00)	100.50 (11.50)	229.5	0.330	0.19	27	98.00 (12.50)	102.00 (14.50)	307.0	0.005*	0.55‡
	Women	RMS_50_ [%RVE]	29	42.68 (17.50)	41.39 (21.15)	166.5	0.270	− 0.20	29	44.19 (24.58)	41.07 (27.67)	136.0	0.127	− 0.28	29	41.50 (27.02)	38.48 (25.35)	190.0	0.552	− 0.11
		MPF [Hz]	29	92.00 (15.00)	90.50 (13.50)	122.5	0.431	− 0.15	29	98.00 (16.75)	97.50 (17.50)	186.0	0.698	− 0.07	29	97.50 (14.50)	96.50 (15.50)	157.5	0.449	− 0.14
Flexor	Men	RMS_50_ [%RVE]	25	20.85 (18.13)	19.07 (16.27)	52.0	0.003*	− 0.59‡	23	18.06 (12.99)	17.14 (17.00)	75.0	0.095	− 0.35†	25	17.27 (12.15)	17.20 (12.26)	146.0	0.657	− 0.09
		MPF [Hz]	25	74.50 (26.50)	78.50 (19.00)	200.0	0.153	0.29	23	78.25 (24.00)	77.50 (17.00)	231.5	0.004*	0.59‡	25	73.25 (22.50)	77.00 (31.50)	264.5	0.006*	0.55‡
	Women	RMS_50_ [%RVE]	25	26.01 (21.29)	23.88 (17.20)	35.0	0.001*	− 0.66‡	24	24.21 (22.52)	24.46 (16.24)	114.0	0.304	− 0.21	23	23.53 (15.73)	20.54 (18.13)	58.0	0.015*	− 0.51‡
		MPF [Hz]	25	73.00 (16.25)	75.50 (21.00)	262.5	0.007*	0.54‡	24	72.00 (10.50)	74.75 (14.38)	235.5	0.003*	0.61‡	23	73.00 (16.00)	77.50 (14.00)	223.0	0.000*	0.78‡
Biceps	Men	RMS_50_ [%RVE]	25	35.38 (20.42)	36.47 (18.25)	200.0	0.313	0.20	26	31.13 (14.74)	37.77 (12.25)	286.0	0.005*	0.55‡	25	37.04 (23.39)	35.52 (30.27)	212.0	0.076	0.35†
		MPF [Hz]	25	60.00 (17.25)	62.00 (16.75)	148.0	0.484	0.14	26	60.25 (12.50)	63.25 (15.50)	237.5	0.012*	0.49†	25	63.50 (12.88)	63.00 (12.25)	175.0	0.474	0.14
	Women	RMS_50_ [%RVE]	29	42.86 (21.61)	35.40 (23.59)	35.0	0.000*	− 0.73‡	26	39.96 (32.71)	34.99 (22.58)	70.0	0.007*	− 0.53‡	28	37.30 (26.44)	32.48 (24.20)	113.0	0.068	− 0.35†
		MPF [Hz]	29	50.00 (8.25)	47.50 (7.38)	106.5	0.028*	− 0.41†	26	49.00 (7.63)	51.00 (7.63)	170.0	0.566	0.11	28	53.25 (9.88)	52.50 (10.75)	256.0	0.226	0.23

*Significant *p* value, *α* = 0.05. †Medium effect size, *r* ≥ 0.3; ^‡^Large effect size, *r* ≥ 0.5. *n* number of subjects in the statistical model; *N* Newton, *IQR* interquartile range, *RMS*_*50*_ median or 50th percentile muscle activity, *RVE* reference voluntary electrical activity, *MPF* median power frequency, *Hz* hertz

#### Electromyographic manifestations of muscle fatigue

For females, the RMS_50_ and MPF of the EXT did not change significantly within days. For males, the RMS_50_ of the EXT significantly decreased within day 2 and the MPF of the EXT significantly increased within day 3. For women, the RMS_50_ of FLEX significantly decreased within days 1 and 3 while the MPF significantly increased, pointing to a recovery of muscle strength [31]. For men, RMS_50_ of the FLEX significantly decreased within day 1 and MPF significantly increased within days 2 and 3. The RMS_50_ and MPF of the BIC significantly increased among men within day 2, pointing to a force increase [31]. Among women, RMS_50_ and MPF both significantly decreased within day 1, pointing to a force decrease [31]. None of the three muscles, EXT, FLEX, and BIC, from which we were able to calculate the RMS_50_ and MPF, showed significant manifestations of muscle fatigue based on one-sample Wilcoxon signed rank tests (Table 2).

### Force differences between men and women

The median maximal force exerted before the experimental task was calculated for each of the four muscles (EXT, FLEX, BIC, TRI) and for both sexes (Table 3). All pre-experimental force levels significantly differed between women and men, with women having significantly lower maximal force levels during the MVCs preceding the experimental task than men.

**Table 3:** Results of the Mann-Whitney test for differences in the maximal force levels between women and men with corresponding effect size *r* (Pearson’s correlation coefficient)

		Median MVC (IQR) [N]		Mann-Whitney test		
Muscle	Day	Men (*n* = 27)	Women (*n* = 30)	*U* value	*p* value	*r*
Extensor	1	180.17 (57.33)	102.25 (35.50)	36.0	0.000*	− 0.78‡
	2	183.00 (54.00)	105.75 (27.42)	38.5	0.000*	− 0.78‡
	3	172.67 (56.50)	105.75 (23.04)	67.0	0.000*	− 0.72‡
Flexor	1	210.50 (100.33)	108.08 (35.71)	30.0	0.000*	− 0.79‡
	2	218.67 (100.83)	124.25 (41.75)	66.0	0.000*	− 0.72‡
	3	231.83 (116.50)	127.92 (50.83)	58.0	0.000*	− 0.73‡
Biceps	1	326.83 (94.33)	181.75 (47.67)	2.0	0.000*	− 0.85‡
	2	332.33 (87.33)	190.75 (47.58)	6.0	0.000*	− 0.84‡
	3	339.67 (79.67)	193.33 (47.58)	10.0	0.000*	− 0.84‡
Triceps	1	255.17 (84.83)	143.58 (35.46)	25.0	0.000*	− 0.80‡
	2	274.00 (92.67)	144.17 (53.08)	49.0	0.000*	− 0.75‡
	3	285.33 (86.67)	158.67 (49.92)	33.0	0.000*	− 0.79‡

*Significant *p* value, *α* = 0.05. ^‡^Large effect size, *r* ≥ 0.5. *MVC* maximal voluntary contraction, *IQR* interquartile range, *n* number of subjects in statistical model, *N* Newton

### Effect of sex on muscle activity

#### Static muscle activity level, RMS_10_

**Fig. 1:**
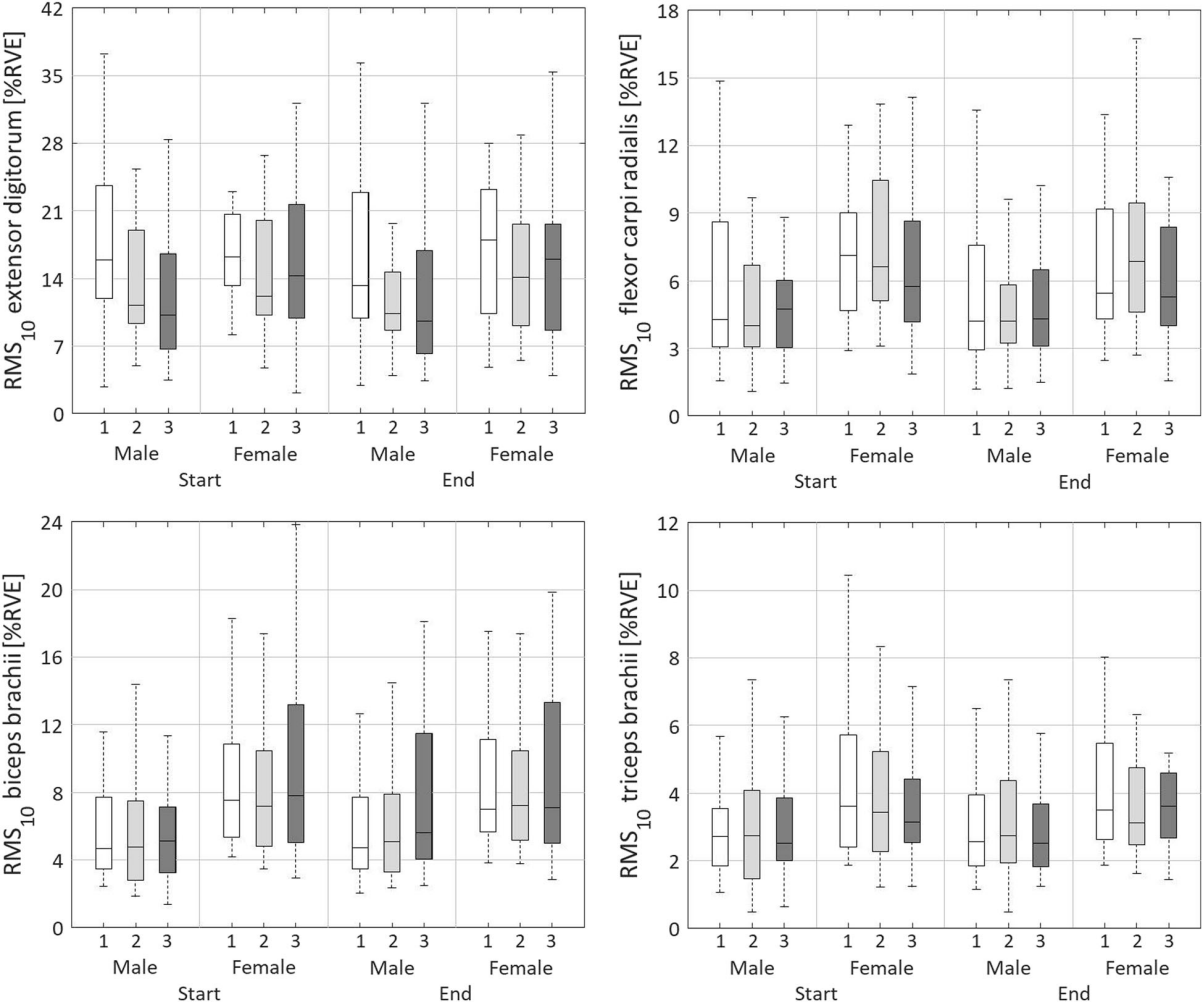
Boxplots representing the static or 10^th^ percentile level of normalised muscle activity (RMS_10_) for the extensor digitorum, flexor carpi radialis, biceps brachii, and triceps bracchii. Boxplots are shown for day 1 (white), day 2 (light grey) and day 3 (dark grey), for males and females, and for start (rows 2 and 3) and end (rows 11 and 12) of the three measurement days

A significant main effect of day was found for RMS_10.DIFF_, and RMS_10.MEAN_ of the EXT (*p* < 0.01; Table 4, Table 5, Fig. 1). RMS_10.DIFF_ decreased more on day 1 compared to days 2 and 3 (*p* < 0.01) and decreased more on day 2 compared to day 3 (*p* < 0.01). The mixed ANOVA also showed a main effect of sex for RMS_10.DIFF_ of the EXT (*p* < 0.05), where men showed a decrease of RMS_10_ and women showed a smaller or no decrease.

**Table 4:** Results of the mixed analysis of variance (ANOVA) for the effect of sex and day on 10^th^ percentile or static muscle activity

Parameter	Muscle	Outcome	Men				Women				Mixed ANOVA					
			*N*	Median (IQR)			*N*	Median (IQR)			Day		Sex		Day × sex	
				Day 1	Day 2	Day 3		Day 1	Day 2	Day 3	*F* value (df_1_, df_2_)	*p* value	*F* value (df_1_, df_2_)	*p* value	*F* value (df_1_, df_2_)	*p* value
RMS_10_	M. extensor digitorum	RMS_10.DIFF_ [%RVE]	27	− 0.93 (4.69)	− 1.59 (5.35)	− 0.30 (4.39)	29	− 0.92 (6.49)	0.22 (4.57)	0.00 (4.13)	60.053 (2, 108)	0.000*	4.028 (1, 54)	0.050*	0.162 (2, 108)	0.851
		RMS_10.MEAN_ [%RVE]	27	14.75 (12.44)	10.51 (7.90)	10.92 (10.39)	29	16.48 (10.70)	13.98 (9.53)	13.25 (11.08)	14.045 (2, 108)	0.000*	0.916 (1, 54)	0.343	0.776 (2, 108)	0.463
	M. flexor carpi radialis	RMS_10.DIFF_ [%RVE]	27	− 0.44 (1.51)	− 0.12 (1.68)	0.00 (1.25)	25	− 0.57 (2.37)	1.29 (3.25)	− 0.29 (1.93)	115.791 (2, 100)	0.000*	0.252 (1, 50)	0.618	0.180 (2, 100)	0.836
		RMS_10.MEAN_ [%RVE]	27	4.30 (6.11)	4.18 (4.21)	4.50 (2.76)	25	6.05 (4.74)	7.00 (5.49)	5.88 (3.89)	1.023 (2, 100)	0.363	5.921 (1, 50)	0.019*	2.590 (2, 100)	0.080
	M. biceps brachii	RMS_10.DIFF_ [%RVE]	26	0.00 (0.76)	0.04 (0.70)	0.21 (1.14)	29	0.36 (1.43)	0.12 (1.17)	0.32 (1.33)	36.615 (2, 106)	0.000*	0.347 (1, 53)	0.559	1.038 (2, 106)	0.358
		RMS_10.MEAN_ [%RVE]	26	4.93 (4.50)	4.91 (3.90)	5.48 (7.64)	29	7.18 (5.08)	6.97 (5.56)	7.54 (8.23)	1.071 (2, 106)	0.346	6.070 (1, 53)	0.017*	0.559 (2, 106)	0.574
	M. triceps brachii	RMS_10.DIFF_ [%RVE]	25	0.00 (0.81)	0.00 (0.35)	0.00 (0.64)	30	0.00 (1.08)	0.00 (0.83)	0.24 (0.66)	55.332 (2, 106)	0.000*	0.876 (1, 53)	0.354	0.263 (2, 106)	0.769
		RMS_10.MEAN_ [%RVE]	25	2.71 (2.07)	2.74 (2.63)	2.57 (1.76)	30	3.40 (3.21)	3.33 (2.80)	3.25 (1.87)	1.237 (2, 106)	0.294	4.495 (1, 53)	0.039*	0.341 (2, 106)	0.712

*Significant *p* value, *α* = 0.05. *N* number of subjects in statistical model, *IQR* interquartile range, *df*_*1*_ degrees of freedom for the number of comparisons within subjects, *df*_*2*_ degrees of freedom for the error term, *RMS*_*10*_ 10^th^ percentile or static muscle activity, *DIFF* difference value between the start (rows 2 and 3) and end (rows 11 and 12) value, *RVE* reference voluntary electrical activity

**Table 5:** Results of the post hoc within-subjects contrasts (day, day × sex) and between-subjects effects (sex) of the mixed analysis of variance (ANOVA) for 10^th^ percentile or static muscle activity with corresponding effect size *r* (Pearson’s correlation coefficient)

Parameter	Muscle	Outcome	Day									Sex			Interaction (day × sex)								
			Day 1 vs. 2			Day 1 vs. 3			Day 2 vs. 3			Men vs. women			Day 1 vs. 2			Day 1 vs. 3			Day 2 vs. 3		
			*F* value (df_1_, df_2_)	*p* value	*r*	*F* value (df_1_, df_2_)	*p* value	*r*	*F* value (df_1_, df_2_)	*p* value	*r*	*F* value (df_1_, df_2_)	*p* value	*r*	*F* value (df_1_, df_2_)	*p* value	*r*	*F* value (df_1_, df_2_)	*p* value	*r*	*F* value (df_1_, df_2_)	*p* value	*r*
RMS_10_	M. extensor digitorum	DIFF	29.22 (1, 54)	0.000*	0.59‡	97.23 (1, 54)	0.000*	0.80‡	39.63 (1, 54)	0.000*	0.65‡	4.03 (1, 54)	0.050*	0.26	0.11 (1, 54)	0.736	0.05	0.05 (1, 54)	0.832	0.03	0.39 (1, 54)	0.534	0.08
		Mean	11.42 (1, 54)	0.001*	0.42†	20.05 (1, 54)	0.000*	0.52‡	6.00 (1, 54)	0.018*	0.32†	0.92 (1, 54)	0.343	0.13	0.53 (1, 54)	0.469	0.10	1.12 (1, 54)	0.295	0.14	0.43 (1, 54)	0.517	0.09
	M. flexor carpi radialis	DIFF	133.94 (1, 50)	0.000*	0.85‡	183.72 (1, 50)	0.000*	0.89‡	0.00 (1, 50)	0.961	0.01	0.25 (1, 50)	0.618	0.07	0.02 (1, 50)	0.881	0.02	0.36 (1, 50)	0.552	0.08	0.22 (1, 50)	0.638	0.07
		Mean	0.07 (1, 50)	0.798	0.04	1.33 (1, 50)	0.255	0.16	1.25 (1, 50)	0.270	0.16	5.92 (1, 50)	0.019*	0.33†	3.84 (1, 50)	0.056	0.27	0.28 (1, 50)	0.598	0.07	4.82 (1, 50)	0.033*	0.30†
	M. biceps brachii	DIFF	36.09 (1, 53)	0.000*	0.64‡	13.39 (1, 53)	0.001*	0.45†	54.41 (1, 53)	0.000*	0.71‡	0.35 (1, 53)	0.559	0.08	2.01 (1, 53)	0.162	0.19	1.75 (1, 53)	0.191	0.18	0.03 (1, 53)	0.873	0.02
		Mean	0.13 (1, 53)	0.723	0.05	0.95 (1, 53)	0.335	0.13	1.55 (1, 53)	0.218	0.17	6.07 (1, 53)	0.017*	0.32†	1.59 (1, 53)	0.214	0.17	0.71 (1, 53)	0.404	0.11	0.02 (1, 53)	0.895	0.02
	M. triceps brachii	DIFF	0.95 (1, 53)	0.335	0.13	149.96 (1, 53)	0.000*	0.86‡	67.21 (1, 53)	0.000*	0.75‡	0.88 (1, 53)	0.354	0.13	0.35 (1, 53)	0.559	0.08	0.53 (1, 53)	0.471	0.10	0.01 (1, 53)	0.922	0.01
		Mean	1.35 (1, 53)	0.251	0.16	2.79 (1, 53)	0.101	0.22	0.08 (1, 53)	0.774	0.04	4.50 (1, 53)	0.039*	0.28	0.00 (1, 53)	0.978	0.00	0.67 (1, 53)	0.418	0.11	0.42 (1, 53)	0.605	0.09

*Significant *p* value, *α* = 0.05. ^†^Medium effect size, *r* ≥ 0.3, ^‡^Large effect size, *r* ≥ 0.5. *df*_*1*_ degrees of freedom for the number of comparisons within subjects, *df*_*2*_ degrees of freedom for the error term, *RMS*_*10*_ 10^th^ percentile or static muscle activity, *DIFF* difference value between the start (rows 2 and 3) and end (rows 11 and 12) value

RMS_10.DIFF_ of the FLEX showed a main effect of day (*p* < 0.01; Table 4, Table 5, Fig. 1). The static muscle activity level decreased more within day 1 compared to days 2 and 3 (*p* < 0.01). Main effects of sex were found for RMS_10.START_ (*p* < 0.05) and RMS_10.MEAN_ (*p* < 0.05) of the FLEX, with women showing higher values than men.

The mixed ANOVA showed a significant main effect of day for RMS_10.DIFF_ of the BIC (*p* < 0.01; Table 4, Table 5, Fig. 1). The static muscle activity level did not change on day 1, whereas it increased on days 2 and 3 (*p* < 0.01); this increase was stronger on day 3 compared to day 2 (*p* < 0.01). A main effect of sex was found for RMS_10.START_ (*p* < 0.05) and RMS_10.MEAN_ (*p* < 0.05) of the BIC, which were both higher for women than for men.

RMS_10.DFF_ of the TRI showed a main effect of day (*p* < 0.01; Table 4, Table 5, Fig. 1). The static muscle activity level increased somewhat more on days 2 and 3 compared to day 1 (*p* < 0.01). A main effect of sex was found for RMS_10.MEAN_ (*p* < 0.05) of the TRI, with values being higher for women than for men.

No main interaction effects between day and sex were found for RMS_10_.

### *Median muscle activity level,* RMS_MEDIAN_

**Fig. 2:**
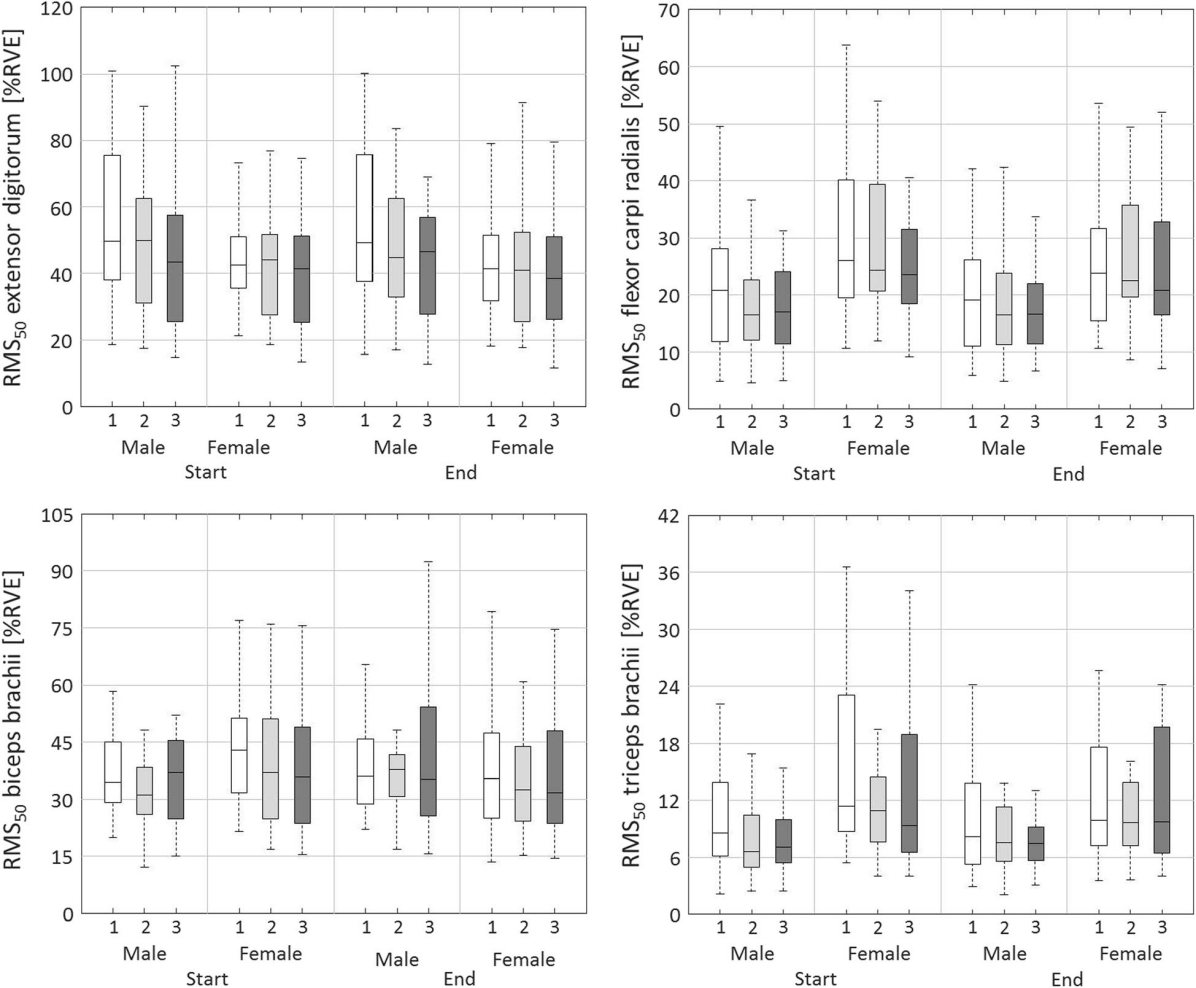
Boxplots representing the median or 50^th^ percentile level of normalised muscle activity (RMS_50_) for the extensor digitorum, flexor carpi radialis, biceps brachii, and triceps bracchii. Boxplots are shown for day 1 (white), day 2 (light grey) and day 3 (dark grey), for males and females, and for start (rows 2 and 3) and end (rows 11 and 12) of the three measurement days

The mixed ANOVA showed a significant main effect of day for RMS_50.DIFF_, and RMS_50.MEAN_ of the EXT (*p* < 0.01; Table 6, Table 7, Fig. 2). RMS_50.MEAN_ was higher on day 1 compared to day 3 (*p* < 0.01) and RMS_MEDIAN_ decreased less on day 3 compared to days 1 and 2 (*p* < 0.01). No main effect of sex for RMS_50_ of the EXT was found.

**Table 6:** Results of the mixed analysis of variance (ANOVA) for the effect of sex and day on 50^th^ percentile or median muscle activity

Parameter	Muscle	Outcome	Men				Women				Statistical analysis (mixed ANOVA)					
			N	Median (IQR)			N	Median (IQR)			Day		Sex		Day × sex	
				Day 1	Day 2	Day 3		Day 1	Day 2	Day 3	*F* value (df_1_, df_2_)	*p* value	*F* value (df_1_, df_2_)	*p* value	*F* value (df_1_, df_2_)	*p* value
RMS_50_	M. extensor digitorum	RMS_50.DIFF_ [%RVE]	27	− 1.49 (8.08)	− 0.81 (4.93)	− 0.85 (5.95)	29	− 0.19 (4.48)	− 0.89 (4.46)	0.18 (5.08)	99.330 (2, 108)	0.000*	0.188 (1, 54)	0.667	0.270 (2, 108)	0.764
		RMS_50.MEAN_ [%RVE]	27	49.79 (38.14)	47.44 (31.48)	45.33 (29.80)	29	42.86 (19.54)	42.83 (27.49)	40.85 (26.98)	6.503 (2, 108)	0.002*	1.366 (1, 54)	0.248	0.761 (2, 108)	0.470
	M. flexor carpi radialis	RMS_50.DIFF_ [%RVE]	27	− 1.70 (6.89)	− 0.87 (2.95)	− 0.15 (3.78)	25	− 2.24 (7.39)	− 1.51 (6.21)	− 1.67 (2.24)	1249.17 (2, 100)	0.000*	0.478 (1, 50)	0.492	1.200 (2, 100)	0.306
		RMS_50.MEAN_ [%RVE]	27	19.51 (16.93)	16.07 (10.77)	17.32 (14.70)	25	24.00 (18.08)	24.03 (20.38)	21.88 (15.24)	1.593 (2, 100)	0.208	4.498 (1, 50)	0.039*	1.498 (2, 100)	0.229
	M. biceps brachii	RMS_50.DIFF_ [%RVE]	26	0.89 (8.30)	4.37 (7.63)	1.20 (8.20)	29	− 6.47 (9.68)	− 3.97 (10.51)	− 3.17 (7.29)	3.539 (2, 106)	0.033*	13.868 (1, 53)	0.000*	0.220 (2, 106)	0.803
		RMS_50.MEAN_ [%RVE]	26	36.07 (18.46)	35.17 (11.54)	35.97 (23.50)	29	37.34 (24.74)	32.83 (23.34)	35.68 (25.52)	3.025 (2, 106)	0.053	0.002 (1, 53)	0.967	0.125 (2, 106)	0.882
	M. triceps brachii	RMS_50.DIFF_ [%RVE]	25	0.00 (2.14)	0.66 (1.74)	0.00 (1.14)	30	− 1.48 (3.94)	− 0.69 (1.94)	0.00 (2.20)	1101.95 (2, 106)	0.000*	5.755 (1, 53)	0.020*	1.669 (2, 106)	0.193
		RMS_50.MEAN_ [%RVE]	25	5.97 (7.65)	5.61 (6.26)	5.62 (2.82)	30	9.59 (5.73)	8.30 (6.06)	7.40 (5.54)	5.046 (2, 106)	0.008*	6.865 (1, 53)	0.011*	0.365 (2, 106)	0.695

*Significant *p* value, *α* = 0.05. *N* number of subjects in statistical model, *IQR* interquartile range, *df*_*1*_ degrees of freedom for the number of comparisons within subjects, *df*_*2*_ degrees of freedom for the error term, *RMS*_*50*_ 50^th^ percentile or median muscle activity, *DIFF* difference value between the start (rows 2 and 3) and end (rows 11 and 12) value, *RVE* reference voluntary electrical activity

**Table 7:** Results of the post hoc within-subjects contrasts (day, day × sex) and between-subjects effects (sex) of the mixed analysis of variance (ANOVA) for 50^th^ percentile or medium muscle activity with corresponding effect size *r* (Pearson’s correlation coefficient)

Parameter	Muscle	Outcome	Day									Sex			Interaction (day × sex)								
			Day 1 vs. 2			Day 1 vs. 3			Day 2 vs. 3			Men vs. women			Day 1 vs. 2			Day 1 vs. 3			Day 2 vs. 3		
			*F* value (df_1_, df_2_)	*p* value	*r*	*F* value (df_1_, df_2_)	*p* value	*r*	*F* value (df_1_, df_2_)	*p* value	*r*	*F* value (df_1_, df_2_)	*p* value	*r*	*F* value (df_1_, df_2_)	*p* value	*r*	*F* value (df_1_, df_2_)	*p* value	*r*	*F* value (df_1_, df_2_)	*p* value	*r*
RMS_50_	M. extensor digitorum	DIFF	0.04 (1, 54)	0.849	0.03	104.45 (1, 54)	0.000*	0.81‡	205.23 (1, 54)	0.000*	0.89‡	0.19 (1, 54)	0.667	0.06	0.65 (1, 54)	0.425	0.11	0.10 (1, 54)	0.756	0.04	0.18 (1, 54)	0.673	0.06
		Mean	4.48 (1, 54)	0.039*	0.28	9.41 (1, 54)	0.003*	0.39†	3.56 (1, 54)	0.065	0.25	1.37 (1, 54)	0.248	0.16	0.08 (1, 54)	0.782	0.04	1.00 (1, 54)	0.323	0.13	1.06 (1,54)	0.307	0.14
	M. flexor carpi radialis	DIFF	865.81 (1, 50)	0.000*	0.97‡	1710.12 (1, 50)	0.000*	0.99‡	739.93 (1, 50)	0.000*	0.97‡	0.48 (1, 50)	0.492	0.10	0.03 (1, 50)	0.862	0.02	1.40 (1, 50)	0.242	0.17	2.89 (1, 50)	0.095	0.23
		Mean	0.02 (1, 50)	0.880	0.02	2.01 (1, 50)	0.163	0.20	2.66 (1, 50)	0.109	0.22	4.50 (1, 50)	0.039*	0.29	2.50 (1, 50)	0.120	0.22	0.00 (1, 50)	0.994	0.00	2.79 (1, 50)	0.101	0.23
	M. biceps brachii	DIFF	1.36 (1, 53)	0.249	0.16	6.68 (1, 53)	0.013*	0.33†	2.37 (1, 53)	0.130	0.21	13.87 (1, 53)	0.000*	0.46†	0.35 (1, 53)	0.559	0.08	0.00 (1, 53)	0.962	0.01	0.33 (1, 53)	0.568	0.08
		Mean	6.45 (1, 53)	0.014*	0.33†	3.27 (1, 53)	0.076	0.24	0.01 (1, 53)	0.920	0.01	0.00 (1, 53)	0.967	0.01	0.01 (1, 53)	0.910	0.02	0.15 (1, 53)	0.696	0.05	0.16 (1, 53)	0.687	0.06
	M. triceps brachii	DIFF	353.58 (1, 53)	0.000*	0.93‡	2005.53 (1, 53)	0.000*	0.99‡	1182.43 (1, 53)	0.000*	0.98‡	5.76 (1, 53)	0.020*	0.31†	0.24 (1, 53)	0.642	0.07	2.92 (1, 53)	0.093	0.23	2.81 (1, 53)	0.099	0.22
		Mean	4.81 (1, 53)	0.033*	0.29	7.79 (1, 53)	0.007*	0.36†	1.07 (1, 53)	0.306	0.14	6.87 (1, 53)	0.011*	0.34†	0.16 (1, 53)	0.695	0.05	0.59 (1, 53)	0.445	0.11	0.27 (1, 53)	0.605	0.07

*Significant *p* value, *α* = 0.05. ^†^Medium effect size, *r* ≥ 0.3; ^‡^Large effect size, *r* ≥ 0.5. *f*_*1*_ degrees of freedom for the number of comparisons within subjects, *df*_*2*_ degrees of freedom for the error term, *RMS*_*50*_ 50th percentile or median muscle activity, *DIFF* difference value between the start (rows 2 and 3) and end (rows 11 and 12) value

A main effect of day was found for RMS_50.DIFF_ of the FLEX (*p* < 0.01; Table 6, Table 7, Fig. 2). The median muscle activity level decreased more within day 1 than within days 2 and 3 (*p* < 0.01) and decreased more within day 2 than within day 3 (*p* < 0.01). There was a main effect of sex for RMS_MEDIAN.MEAN_ (*p* < 0.05) of the FLEX, with values for females being higher than for males.

A main effect of day was found for RMS_50.DIFF_ (*p* < 0.05) of the BIC (Table 6, Table 7, Fig. 2). The median muscle activity level decreased within day 1 whereas it remained unchanged within day 3 (*p* < 0.05). A main effect of sex was found for RMS_50.DIFF_ of the BIC (*p* < 0.01), with women showing a decreased and men an increased RMS_50_ within days.

Main effects of day were found for RMS_50.DIFF_, and RMS_50.MEAN_ (*p* < 0.01; Table 6, Table 7, Fig. 2) of the TRI. RMS_50.MEAN_ was higher on day 1 compared to day 3 (*p* < 0.05). The median muscle activity level decreased most within day 1, then in day 2 and remained stable within day 3 (*p* < 0.01). There were main effects of sex for RMS_50.START_ (*p* < 0.01), RMS_50.DIFF_ (*p* < 0.01), and RMS_50.MEAN_ (*p* < 0.01) of the TRI. Women had a higher RMS_50.MEAN_ across days than men, and women showed a decrease of RMS_50_ within days compared to an increase or no change among men.

No main interaction effects between day and sex were found for RMS_50_.

### Peak muscle activity level, RMS_90_

**Fig. 3:**
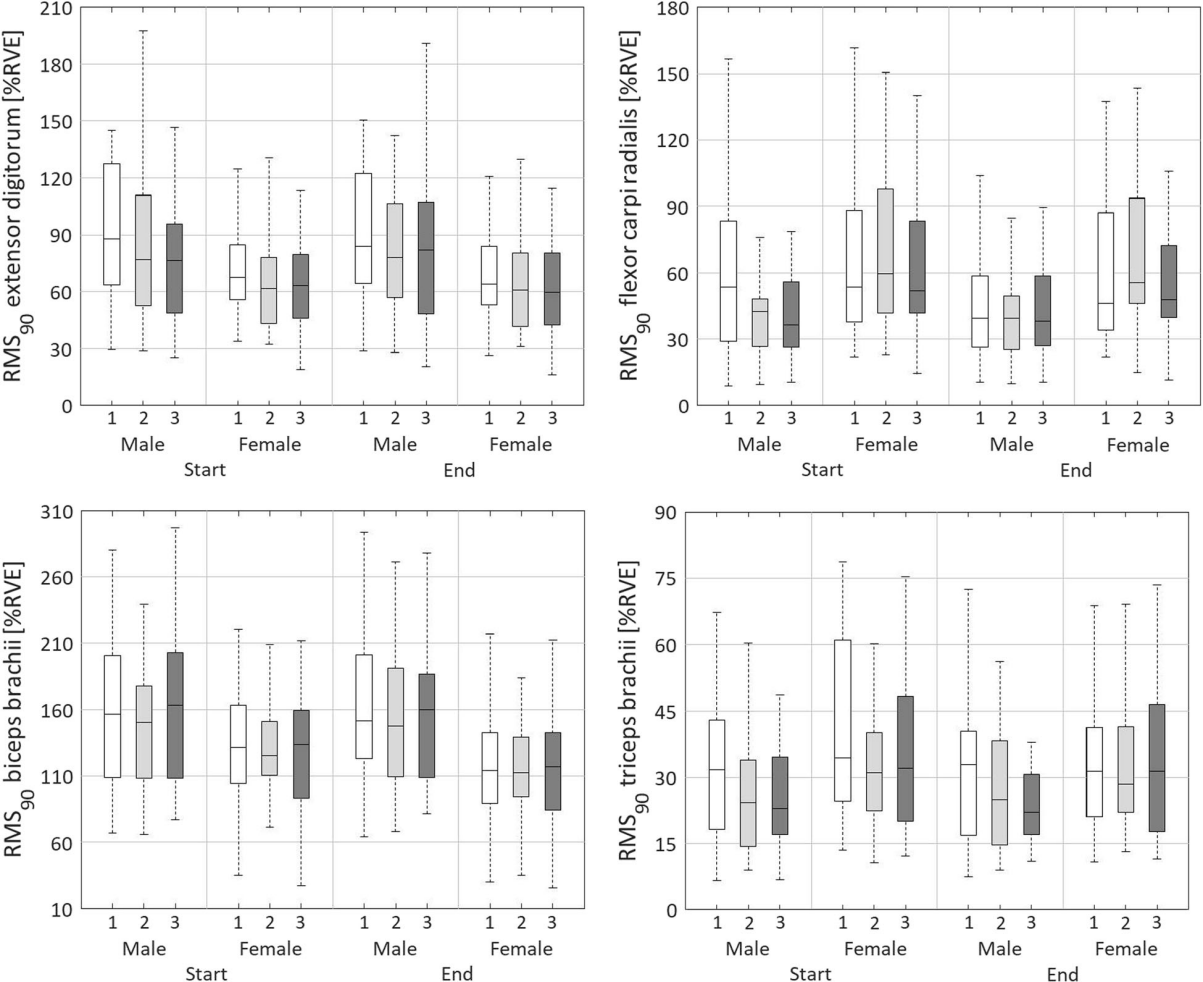
Boxplots representing the peak or 90^th^ percentile level of normalised muscle activity (RMS_90_) for the extensor digitorum, flexor carpi radialis, biceps brachii, and triceps bracchii. Boxplots are shown for day 1 (white), day 2 (light grey) and day 3 (dark grey), for males and females, and for start (rows 2 and 3) and end (rows 11 and 12) of the three measurement days

Main effects of day were found for RMS_90.DIFF_, and RMS_90.MEAN_ of the EXT (*p* < 0.01; Table 8, Table 9, Fig. 3). RMS_90.MEAN_ was higher on day 1 than day 3 (*p* < 0.05). The peak muscle activity significantly decreased most on day 1, followed by day 3 and day 2 (*p* < 0.01). We found a main effect of sex for RMS_90.MEAN_ (*p* < 0.05) of the EXT, with men showing higher RMS_90.MEAN_ than women.

**Table 8:** Results of the mixed analysis of variance (ANOVA) for the effect of sex and day on 90^th^ percentile or peak muscle activity

Parameter	Muscle	Outcome	Men				Women				Statistical analysis (mixed ANOVA)					
			*N*	Median (IQR)			*N*	Median (IQR)			Day		Sex		Day × sex	
				Day 1	Day 2	Day 3		Day 1	Day 2	Day 3	*F* value (df_1_, df_2_)	*p* value	*F* value (df_1_, df_2_)	*p* value	*F* value (df_1_, df_2_)	*p* value
RMS_90_	M. extensor digitorum	RMS_90.DIFF_ [%RVE]	27	− 2.68 (14.96)	− 0.87 (12.57)	− 1.59 (8.93)	29	− 0.39 (6.94)	− 1.10 (8.76)	− 1.41 (8.99)	158.116 (2, 108)	0.000*	1.223 (1, 54)	0.274	0.445 (2, 108)	0.642
		RMS_90.MEAN_ [%RVE]	27	83.56 (55.95)	81.18 (57.21)	79.02 (61.00)	29	63.94 (27.19)	59.16 (38.22)	60.43 (39.22)	5.203 (2, 108)	0.007*	4.213 (1, 54)	0.045*	0.791 (2, 108)	0.456
	M. flexor carpi radialis	RMS_90.DIFF_ [%RVE]	27	− 3.47 (19.29)	0.42 (9.05)	1.23 (8.72)	25	− 4.48 (19.18)	− 1.60 (20.11)	− 4.61 (10.96)	1286.88 (2, 100)	0.000*	0.427 (1, 50)	0.516	3.929 (2, 100)	0.023*
		RMS_90.MEAN_ [%RVE]	27	44.12 (44.97)	38.53 (22.64)	38.09 (29.19)	25	53.81 (52.24)	56.28 (59.29)	48.84 (40.93)	0.757 (2, 100)	0.472	2.758 (1, 50)	0.103	2.199 (2, 100)	0.116
	M. biceps brachii	RMS_90.DIFF_ [%RVE]	26	− 4.08 (18.65)	− 3.58 (28.77)	− 2.93 (32.07)	29	− 12.04 (25.00)	− 14.55 (16.31)	− 12.07 (20.59)	66.653 (2, 106)	0.000*	10.381 (1, 53)	0.002*	1.634 (2, 106)	0.200
		RMS_90.MEAN_ [%RVE]	26	150.59 (87.50)	143.73 (86.54)	165.71 (100.66)	29	125.75 (58.65)	118.82 (37.84)	124.55 (60.00)	0.981 (2, 106)	0.378	5.539 (1, 53)	0.022*	0.345 (2, 106)	0.709
	M. triceps brachii	RMS_90.DIFF_ [%RVE]	25	0.76 (5.29)	0.00 (6.55)	− 1.09 (5.28)	30	− 4.39 (9.25)	− 1.43 (5.73)	− 0.69 (5.50)	816.754 (2, 106)	0.000*	7.136 (1, 53)	0.010*	2.657 (2, 106)	0.075
		RMS_90.MEAN_ [%RVE]	25	30.86 (24.85)	23.86 (20.70)	22.19 (14.07)	30	32.06 (24.27)	30.41 (20.36)	30.97 (27.62)	4.173 (2, 106)	0.018*	3.278 (1, 53)	0.076	0.056 (2, 106)	0.945

*Significant *p* value, *α* = 0.05. *N* number of subjects in statistical model, *IQR* interquartile range, *df*_*1*_ degrees of freedom for the number of comparisons within subjects, *df*_*2*_ degrees of freedom for the error term, *RMS*_*90*_ 90th percentile or peak muscle activity, *DIFF* difference value between the start (rows 2 and 3) and end (rows 11 and 12) value, *RVE* reference voluntary electrical activity

**Table 9:** Results of the post hoc within-subjects contrasts (day, day × sex) and between-subjects effects (sex) of the mixed analysis of variance (ANOVA) for 90^th^ percentile or peak muscle activity with corresponding effect size *r* (Pearson’s correlation coefficient)

Parameter	Muscle	Outcome	Day									Sex			Interaction (day × sex)								
			Day 1 vs. 2			Day 1 vs. 3			Day 2 vs. 3			Men vs. women			Day 1 vs. 2			Day 1 vs. 3			Day 2 vs. 3		
			*F* value (df_1_, df_2_)	*p* value	*r*	*F* value (df_1_, df_2_)	*p* value	*r*	*F* value (df_1_, df_2_)	*p* value	*r*	*F* value (df_1_, df_2_)	*p* value	*r*	*F* value (df_1_, df_2_)	*p* value	*r*	*F* value (df_1_, df_2_)	*p* value	*r*	*F* value (df_1_, df_2_)	*p* value	*r*
RMS_90_	M. extensor digitorum	DIFF	378.10 (1, 54)	0.000*	0.94‡	75.54 (1, 54)	0.000*	0.76‡	70.51 (1, 54)	0.000*	0.75‡	1.22 (1, 54)	0.247	0.15	0.19 (1, 54)	0.663	0.06	0.24 (1, 54)	0.630	0.07	0.95 (1, 54)	0.334	0.13
		Mean	3.34 (1, 54)	0.073	0.24	7.98 (1, 54)	0.007*	0.36†	2.82 (1, 54)	0.099	0.22	4.21 (1, 54)	0.045*	0.27	0.15 (1, 54)	0.696	0.05	1.14 (1, 54)	0.290	0.14	0.84 (1, 54)	0.363	0.12
	M. flexor carpi radialis	DIFF	1329.46 (1, 50)	0.000*	0.98‡	1545.98 (1, 50)	0.000*	0.98‡	787.08 (1, 50)	0.000*	0.97‡	0.43 (1, 50)	0.516	0.09	1.92 (1, 50)	0.172	0.19	4.55 (1, 50)	0.038*	0.29	3.75 (1, 50)	0.059	0.26
		Mean	0.02 (1, 50)	0.882	0.02	0.69 (1, 50)	0.411	0.12	1.88 (1, 50)	0.176	0.19	2.76 (1, 50)	0.103	0.23	3.89 (1, 50)	0.054	0.27	0.01 (1, 50)	0.914	0.02	4.51 (1, 50)	0.039*	0.29
	M. biceps brachii	DIFF	71.93 (1, 53)	0.000*	0.76‡	95.50 (1, 53)	0.000*	0.80‡	4.84 (1, 53)	0.032*	0.29	10.38 (1, 53)	0.002*	0.40†	2.40 (1, 53)	0.127	0.21	1.79 (1, 53)	0.838	0.18	0.04 (1, 53)	0.838	0.03
		Mean	2.70 (1, 53)	0.106	0.22	0.61 (1, 53)	0.437	0.11	0.26 (1, 53)	0.614	0.07	5.54 (1, 53)	0.022*	0.31†	0.00 (1, 53)	0.980	0.00	0.42 (1, 53)	0.521	0.09	0.49 (1, 53)	0.486	0.10
	M. triceps brachii	DIFF	333.71 (1, 53)	0.000*	0.93‡	1488.00 (1, 53)	0.000*	0.98‡	674.81 (1, 53)	0.000*	0.96‡	7.14 (1, 53)	0.010*	0.34†	0.17 (1, 53)	0.678	0.06	4.39 (1, 53)	0.041*	0.28	5.42 (1, 53)	0.024*	0.30†
		Mean	1.96 (1, 53)	0.167	0.19	10.00 (1, 53)	0.003*	0.40†	1.92 (1, 53)	0.171	0.19	3.28 (1, 53)	0.076	0.24	0.05 (1, 53)	0.818	0.03	0.00 (1, 53)	0.965	0.01	0.12 (1, 53)	0.734	0.05

*Significant *p* value, *α* = 0.05. ^†^Medium effect size, *r* ≥ 0.3; ^‡^Large effect size, *r* ≥ 0.5. *df*_*1*_ degrees of freedom for the number of comparisons within subjects, *df*_*2*_ degrees of freedom for the error term, *RMS*_*90*_ 90th percentile or peak muscle activity, *DIFF* difference value between the start (rows 2 and 3) and end (rows 11 and 12) value

For the FLEX, a main effect of day was found for RMS_90.DIFF_ (*p* < 0.01; Table 8, Table 9, Fig. 3). The peak muscle activity level significantly decreased most within day 1, followed by day 3 and day 2 (*p* < 0.01). No main effect of sex was found for RMS_90_ of the FLEX. A significant day × sex interaction effect was found for RMS_90.DIFF_ of the FLEX (*p* < 0.05). Within days 1 and 2, the peak muscle activity increased slightly more within males compared to females, whereas within day 3, this pattern was reversed (*p* < 0.05).

RMS_90.DIFF_ of the BIC showed a significant main effect of day (*p* < 0.01; Table 8, Table 9, Fig. 3). The peak muscle activity level decreased on all 3 days, but slightly stronger within day 1 compared to day 3 (*p* < 0.01), and stronger within day 2 compared to day 1 (*p* < 0.01). A main effect of sex was found for RMS_90.DIFF_ (*p* < 0.01) and RMS_90.MEAN_ (*p* < 0.05), where women had lower mean values and stronger decreases within days than men.

The mixed ANOVA showed a main effect of day for RMS_90.DIFF_ (*p* < 0.01), and RMS_90.MEAN_ (*p* < 0.05) of the TRI (Table 8, Table 9, Fig. 3). RMS_90.MEAN_ was higher on day 1 than day 3 (*p* < 0.01). The peak muscle activity level decreased more within day 1 compared to days 2 and 3 (*p* < 0.01) and decreased more within day 3 compared to day 2 (*p* < 0.01). A main effect of sex was found for RMS_90.DIFF_ (*p* < 0.05) of the TRI. Women showed stronger decreases of RMS_90_ than men.

### Effect of sex on motor variability

#### Absolute cycle-to-cycle variability of muscle activity, RMS_SD_

**Fig. 4:**
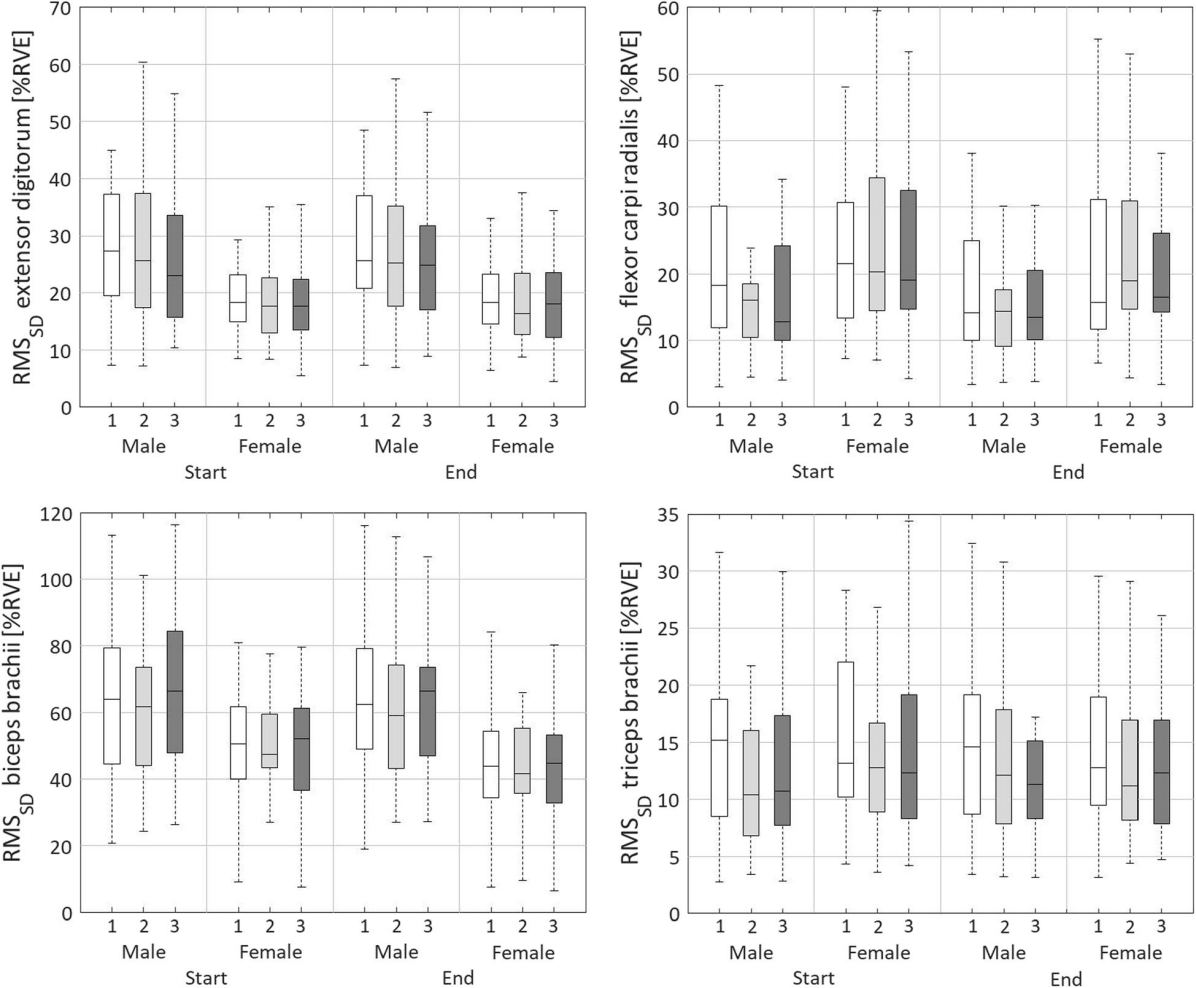
Boxplots representing the absolute variability as the pooled cycle-to-cycle standard deviation of muscle activity (RMS_SD_) for the extensor digitorum, flexor carpi radialis, biceps brachii, and triceps bracchii. Boxplots are shown for day 1 (white), day 2 (light grey) and day 3 (dark grey), for males and females, and for start (rows 2 and 3) and end (rows 11 and 12) of the three measurement days

A main effect of day was found for RMS_SD.DIFF_ of the EXT (*p* < 0.01; Table 10, Table 11, Fig. 4). Absolute variability decreased more on day 1 than on days 2 and 3 (*p* < 0.01). There was a main effect of sex for RMS_SD.START_ (*p* < 0.01) and RMS_SD.MEAN_ (F*p* < 0.01) of the EXT. Both RMS_SD.START_ and RMS_SD.MEAN_ were lower for women than for men.

**Table 10:** Results of the mixed analysis of variance (ANOVA) for the effect of sex and day on absolute cycle-to-cycle variability of muscle activity

Parameter	Muscle	Outcome	Men				Women				Statistical analysis (mixed ANOVA)					
			*N*	Median (IQR)			*N*	Median (IQR)			Day		Sex		Day × sex	
				Day 1	Day 2	Day 3		Day 1	Day 2	Day 3	*F* value (df_1_, df_2_)	*p* value	*F* value (df_1_, df_2_)	*p* value	*F* value (df_1_, df_2_)	*p* value
RMS_SD_	M. extensor digitorum	RMS_SD.START_ [%RVE]	27	27.34 (19.33)	25.56 (21.61)	23.07 (18.99)	29	18.38 (9.08)	17.67 (9.82)	17.70 (8.99)	2.195 (2, 108)	0.116	7.621 (1, 54)	0.008*	0.509 (2, 108)	0.603
		RMS_SD.DIFF_ [%RVE]	27	− 0.02 (6.69)	− 0.19 (5.55)	0.74 (4.79)	29	− 0.23 (3.27)	− 0.06 (2.61)	− 0.72 (3.72)	103.283 (2, 108)	0.000*	0.041 (1, 54)	0.841	0.361 (2, 108)	0.698
		RMS_SD.MEAN_ [%RVE]	27	28.03 (17.61)	25.88 (19.00)	23.89 (20.30)	29	18.43 (7.46)	17.10 (10.63)	17.94 (9.01)	2.013 (2, 108)	0.139	8.159 (1, 54)	0.006*	0.447 (2, 108)	0.640
	M. flexor carpi radialis	RMS_SD.START_ [%RVE]	27	18.30 (18.50)	16.04 (8.49)	12.79 (15.11)	25	21.49 (17.69)	20.28 (20.42)	19.01 (18.42)	0.427 (2, 100)	0.654	1.158 (1, 50)	0.287	1.316 (2, 100)	0.273
		RMS_SD.DIFF_ [%RVE]	27	− 1.67 (6.54)	− 0.08 (3.03)	− 0.25 (3.46)	25	− 1.96 (8.08)	− 0.77 (7.14)	− 1.40 (4.03)	1462.34 (2, 100)	0.000*	0.469 (1, 50)	0.497	2.784 (2, 100)	0.067
		RMS_SD.MEAN_ [%RVE]	27	15.95 (16.77)	14.62 (7.34)	13.57 (15.33)	25	19.00 (19.51)	20.52 (21.00)	16.91 (16.73)	0.528 (2, 100)	0.592	1.141 (1, 50)	0.291	2.070 (2, 100)	0.132
	M. biceps brachii	RMS_SD.START_ [%RVE]	26	64.06 (36.24)	61.73 (30.25)	66.36 (37.99)	29	50.50 (22.99)	47.46 (16.60)	52.08 (25.65)	0.590 (2, 106)	0.556	4.148 (1, 53)	0.047*	0.868 (2, 106)	0.423
		RMS_SD.DIFF_ [%RVE]	26	− 1.61 (8.24)	− 1.30 (9.77)	− 0.71 (12.76)	29	− 4.25 (10.01)	− 6.48 (7.01)	− 5.22 (8.85)	30.392 (2, 106)	0.000*	12.311 (1, 53)	0.001*	1.193 (2, 106)	0.307
		RMS_SD.MEAN_ [%RVE]	26	61.07 (32.50)	58.62 (33.84)	67.08 (34.30)	29	47.10 (20.47)	45.51 (16.76)	47.17 (21.32)	0.846 (2, 106)	0.432	7.396 (1, 53)	0.009*	0.622 (2, 106)	0.539
	M. triceps brachii	RMS_SD.START_ [%RVE]	25	15.15 (10.36)	10.41 (9.42)	10.71 (10.00)	30	13.17 (12.20)	12.79 (8.02)	12.30 (11.07)	3.649 (2, 106)	0.029*	0.902 (1, 53)	0.347	0.097 (2, 106)	0.908
		RMS_SD.DIFF_ [%RVE]	25	0.56 (4.40)	0.13 (3.69)	0.05 (2.63)	30	− 1.78 (3.16)	− 1.07 (3.08)	− 0.14 (2.56)	454.218 (2, 106)	0.000*	10.274 (1, 53)	0.002*	3.938 (2, 106)	0.022*
		RMS_SD.MEAN_ [%RVE]	25	14.84 (11.04)	12.10 (10.26)	11.54 (8.55)	30	13.55 (10.41)	12.01 (8.92)	12.83 (9.87)	2.484 (2, 106)	0.088	0.224 (1, 53)	0.638	0.019 (2, 106)	0.981

*Significant *p* value, *α* = 0.05. *N* number of subjects in statistical model, *IQR* interquartile range, *df*_*1*_ degrees of freedom for the number of comparisons within subjects, *df*_*2*_ degrees of freedom for the error term, *RMS*_*SD*_ absolute cycle-to-cycle variability of muscle activity, *START* initial value, *DIFF* difference value between the start (rows 2 and 3) and end (rows 11 and 12) value, *RVE* reference voluntary electrical activity

**Table 11:** Results of the post hoc within-subjects contrasts (day, day × sex) and between-subjects effects (sex) of the mixed analysis of variance (ANOVA) for absolute cycle-to-cycle variability of muscle activity with corresponding effect size *r* (Pearson’s correlation coefficient)

Parameter	Muscle	Outcome	Day									Sex			Interaction (day × sex)								
			Day 1 vs. 2			Day 1 vs. 3			Day 2 vs. 3			Men vs. women			Day 1 vs. 2			Day 1 vs. 3			Day 2 vs. 3		
			*F* value (df_1_, df_2_)	*p* value	*r*	*F* value (df_1_, df_2_)	*p* value	*r*	*F* value (df_1_, df_2_)	*p* value	*r*	*F* value (df_1_, df_2_)	*p* value	*r*	*F* value (df_1_, df_2_)	*p* value	*r*	*F* value (df_1_, df_2_)	*p* value	*r*	*F* value (df_1_, df_2_)	*p* value	*r*
RMS_SD_	M. extensor digitorum	START	2.46 (1, 54)	0.123	0.21	3.38 (1, 54)	0.072	0.24	0.39 (1, 54)	0.537	0.08	7.62 (1, 54)	0.008*	0.35†	0.11 (1, 54)	0.737	0.05	0.79 (1, 54)	0.378	0.12	0.49 (1, 54)	0.485	0.10
		DIFF	246.00 (1, 54)	0.000*	0.91‡	114.98 (1, 54)	0.000*	0.82‡	0.02 (1, 54)	0.091	0.02	0.04 (1, 54)	0.841	0.03	0.06 (1, 54)	0.800	0.03	0.28 (1, 54)	0.598	0.07	0.65 (1, 54)	0.425	0.11
		Mean	1.47 (1, 54)	0.230	0.16	3.38 (1, 54)	0.072	0.24	0.81 (1, 54)	0.371	0.12	8.16 (1, 54)	0.006*	0.36†	0.07 (1, 54)	0.794	0.04	0.70 (1, 54)	0.406	0.11	0.47 (1, 54)	0.498	0.09
	M. flexor carpi radialis	START	0.19 (1, 50)	0.662	0.06	0.57 (1, 50)	0.453	0.10	0.40 (1, 50)	0.528	0.09	1.16 (1, 50)	0.287	0.15	2.90 (1, 50)	0.095	0.23	0.14 (1, 50)	0.711	0.05	1.91 (1, 50)	0.173	0.19
		DIFF	1474.13 (1, 50)	0.000*	0.98‡	1747.90 (1, 50)	0.000*	0.99‡	890.72 (1, 50)	0.000*	0.97‡	0.47 (1, 50)	0.497	0.09	2.43 (1, 50)	0.125	0.21	3.32 (1, 50)	0.074	0.24	1.92 (1, 50)	0.172	0.19
		Mean	0.00 (1, 50)	0.993	0.00	0.55 (1, 50)	0.460	0.10	1.26 (1, 50)	0.268	0.15	1.14 (1, 50)	0.291	0.15	3.41 (1, 50)	0.071	0.25	0.00 (1, 50)	0.975	0.00	4.87 (1, 50)	0.003*	0.29
	M. biceps brachii	START	1.79 (1, 53)	0.186	0.18	0.33 (1, 53)	0.566	0.08	0.18 (1, 53)	0.675	0.06	4.15 (1, 53)	0.047*	0.27	0.77 (1, 53)	0.384	0.12	0.32 (1, 53)	0.577	0.08	1.47 (1, 53)	0.231	0.16
		DIFF	31.04 (1, 53)	0.000*	0.61‡	44.48 (1, 53)	0.000*	0.68‡	4.24 (1, 53)	0.044*	0.27	12.31 (1, 53)	0.001*	0.43†	2.02 (1, 53)	0.161	0.19	1.09 (1, 53)	0.301	0.14	0.12 (1, 53)	0.727	0.05
		Mean	2.32 (1, 53)	0.134	0.20	0.33 (1, 53)	0.567	0.08	0.42 (1, 53)	0.521	0.09	7.40 (1, 53)	0.009*	0.35†	0.02 (1, 53)	0.881	0.02	0.69 (1, 53)	0.410	0.11	0.94 (1, 53)	0.336	0.13
	M. triceps brachii	START	2.29 (1, 53)	0.136	0.20	7.19 (1, 53)	0.010*	0.35†	1.21 (1, 53)	0.275	0.15	0.90 (1, 53)	0.347	0.13	0.09 (1, 53)	0.760	0.04	0.17 (1, 53)	0.686	0.06	0.00 (1, 53)	0.954	0.01
		DIFF	279.60 (1, 53)	0.000*	0.92‡	889.84 (1, 53)	0.000*	0.97‡	156.86 (1, 53)	0.000*	0.86‡	10.27 (1, 53)	0.002*	0.40†	0.40 (1, 53)	0.530	0.09	7.48 (1, 53)	0.008*	0.35†	6.14 (1, 53)	0.016*	0.32†
		Mean	1.45 (1, 53)	0.233	0.16	5.62 (1, 53)	0.021*	0.31†	0.81 (1, 53)	0.371	0.12	0.22 (1, 53)	0.638	0.06	0.00 (1, 53)	0.946	0.01	0.04 (1, 53)	0.835	0.03	0.02 (1, 53)	0.898	0.02

*Significant *p* value, *α* = 0.05. ^†^Medium effect size, *r* ≥ 0.3; ^‡^Large effect size, *r* ≥ 0.5. *df*_*1*_ degrees of freedom for the number of comparisons within subjects, *df*_*2*_ degrees of freedom for the error term, *RMS*_*SD*_ absolute cycle-to-cycle variability of muscle activity, *START* initial value, *DIFF* difference value between the start (rows 2 and 3) and end (rows 11 and 12) value

RMS_SD.DIFF_ of the FLEX showed a main effect of day (*p* < 0.01; Table 10, Table 11, Fig. 4). The absolute variability decreased most within day 1, followed by day 3 and day 2 (*p* < 0.01). No main effect of sex was found for RMS_SD_ of the FLEX.

The mixed ANOVA showed a main effect of day for RMS_SD.DIFF_ of the BIC (*p* < 0.01; Table 10, Table 11, Fig. 4). The absolute variability decreased within days, within days 2 and 3 somewhat more than within day 1 (*p* < 0.01). Main effects of sex were found for RMS_SD.START_ (*p* < 0.05), RMS_SD.DIFF_ (*p* < 0.01), and RMS_SD.MEAN_ (*p* < 0.01) of the BIC. Women had a lower RMS_SD.START_ and RMS_SD.MEAN_ than males and showed a stronger decrease of RMS_SD_ within days than males.

The mixed ANOVA revealed a main effect of day for RMS_SD.START_ (*p* < 0.05) and RMS_SD.DIFF_ (*p* < 0.01) of the TRI (Table 10, Table 11, Fig. 4). RMS_SD.START_ was higher on day 1 compared to day 3 (*p* < 0.05). The absolute variability decreased most within day 1, then day 2 and then day 3 (*p* < 0.01). RMS_SD.DIFF_ of the TRI also showed a main effect of sex (*p* < 0.01), with men showing an increase and women a decrease of RMS_SD_ within days. A main interaction effect of day × sex was found for RMS_SD.DIFF_ (*p* < 0.05). Within days 1 and 2, the absolute variability decreased for females and increased for males, whereas on day 3, it did not differ between both sexes.

#### Relative cycle-to-cycle variability of muscle activity, RMS_CV_

**Fig. 5:**
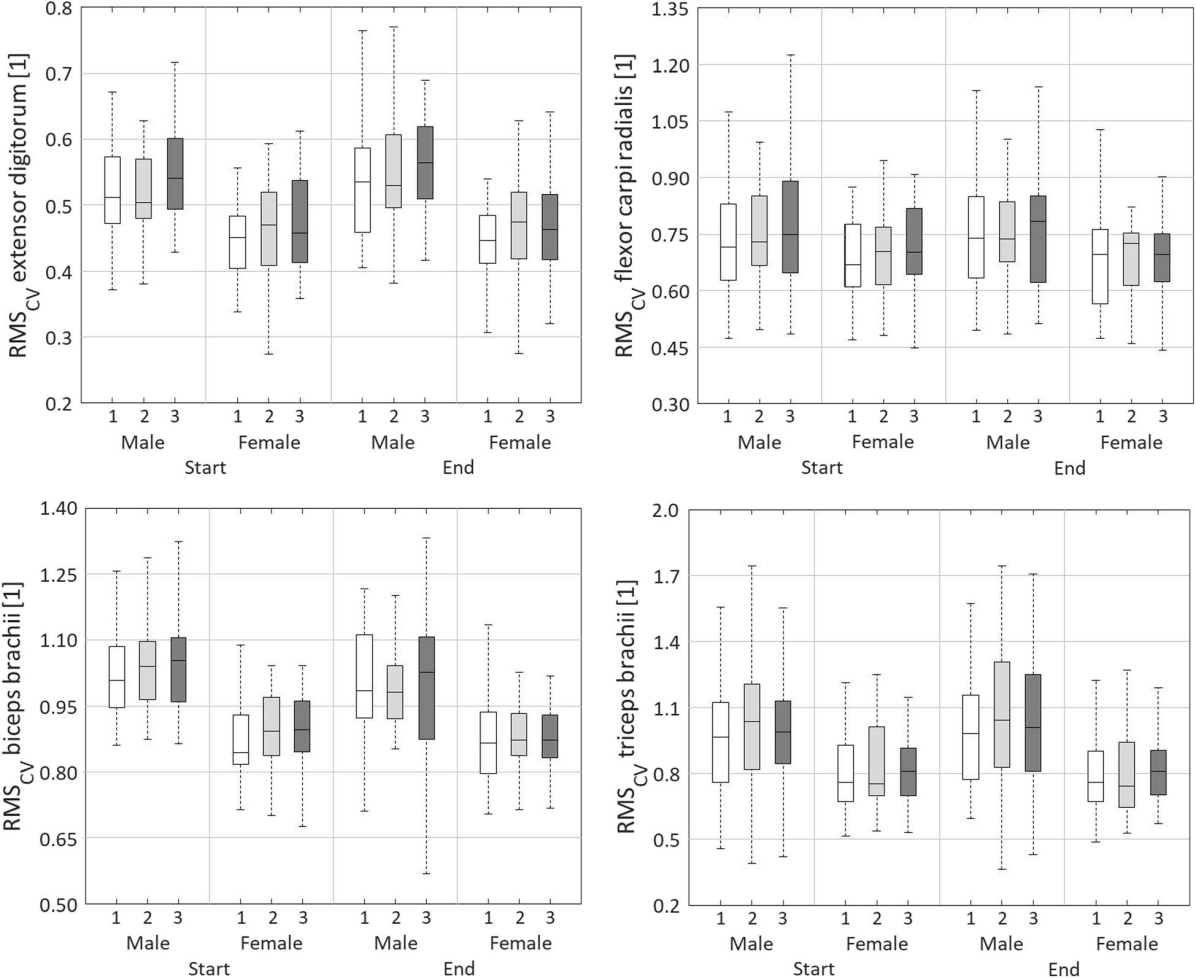
Boxplots representing the relative variability or coefficient of variation as the pooled cycle-to-cycle standard deviation devided by the mean of muscle activity (RMS_CV_) for the extensor digitorum, flexor carpi radialis, biceps brachii, and triceps bracchii. Boxplots are shown for day 1 (white), day 2 (light grey) and day 3 (dark grey), for males and females, and for start (rows 2 and 3) and end (rows 11 and 12) of the three measurement days

Main effects of day were found for RMS_CV.START_, RMS_CV.DIFF_, and RMS_CV.MEAN_ of the EXT (Table 12, Table 13, Fig. 5). RMS_CV.START_ was higher on day 3 compared to day 1 (*p* < 0.05). Similarly, RMS_CV.MEAN_ was higher on day 3 than day 1 (*p* < 0.01). The relative variability did not change within day 1 compared to an increase within day 2 (*p* < 0.01) and a decrease within day three (*p* < 0.01). There was a main effect of sex for RMS_CV.START_ (*p* < 0.01) and RMS_CV.MEAN_ (*p* < 0.01) of the EXT, both being higher for women than for men.

**Table 12:** Results of the mixed analysis of variance (ANOVA) for the effect of sex and day on relative cycle-to-cycle variability of muscle activity

Parameter	Muscle	Outcome	Men				Women				Statistical analysis (mixed ANOVA)					
			*N*	Median (IQR)			*N*	Median (IQR)			Day		Sex		Day × sex	
				Day 1	Day 2	Day 3		Day 1	Day 2	Day 3	*F* value (df_1_, df_2_)	*p* value	*F* value (df_1_, df_2_)	*p* value	*F* value (df_1_, df_2_)	*p* value
RMS_CV_	M. extensor digitorum	RMS_CV.START_ [1]	27	0.51 (0.10)	0.50 (0.10)	0.54 (0.11)	29	0.45 (0.08)	0.47 (0.11)	0.46 (0.13)	5.180 (2, 108)	0.007*	14.750 (1, 54)	0.000*	0.226 (2, 108)	0.798
		RMS_CV.DIFF_ [1]	27	0.00 (0.09)	0.01 (0.07)	0.00 (0.08)	29	0.01 (0.06)	0.01 (0.04)	− 0.01 (0.08)	24.453 (2, 108)	0.000*	0.985 (1, 54)	0.325	0.028 (2, 108)	0.972
		RMS_CV.MEAN_ [1]	27	0.53 (0.09)	0.53 (0.08)	0.54 (0.10)	29	0.45 (0.07)	0.46 (0.11)	0.45 (0.10)	5.231 (2, 108)	0.007*	19.067 (1, 54)	0.000*	0.125 (2, 108)	0.882
	M. flexor carpi radialis	RMS_CV.START_ [1]	27	0.72 (0.22)	0.73 (0.19)	0.75 (0.26)	25	0.67 (0.17)	0.70 (0.16)	0.70 (0.19)	2.082 (2, 100)	0.130	2.704 (1, 50)	0.106	0.025 (2, 100)	0.975
		RMS_CV.DIFF_ [1]	27	0.01 (0.07)	0.00 (0.07)	− 0.01 (0.12)	25	− 0.01 (0.09)	− 0.01 (0.06)	− 0.02 (0.07)	95.381 (2, 100)	0.000*	0.141 (1, 50)	0.709	0.095 (2, 100)	0.910
		RMS_CV.MEAN_ [1]	27	0.74 (0.21)	0.75 (0.20)	0.79 (0.23)	25	0.69 (0.16)	0.72 (0.15)	0.70 (0.19)	0.358 (2, 100)	0.700	3.455 (1, 50)	0.069	0.299 (2, 100)	0.742
	M. biceps brachii	RMS_CV.START_ [1]	26	1.01 (0.15)	1.04 (0.15)	1.05 (0.15)	29	0.84 (0.12)	0.89 (0.14)	0.90 (0.12)	3.882 (2, 106)	0.024*	7.648 (1, 53)	0.008*	0.875 (2, 106)	0.420
		RMS_CV.DIFF_ [1]	26	− 0.02 (0.06)	− 0.03 (0.09)	− 0.01 (0.07)	29	0.01 (0.05)	− 0.03 (0.03)	− 0.03 (0.05)	114.189 (2, 106)	0.000*	0.493 (1, 53)	0.486	0.290 (2, 106)	0.748
		RMS_CV.MEAN_ [1]	26	1.01 (0.17)	1.01 (0.12)	1.03 (0.19)	29	0.86 (0.15)	0.88 (0.11)	0.89 (0.11)	1.015 (2, 106)	0.366	7.583 (1, 53)	0.008*	0.929 (2, 106)	0.398
	M. triceps brachii	RMS_CV.START_ [1]	25	0.96 (0.37)	1.04 (0.42)	0.99 (0.33)	30	0.76 (0.27)	0.75 (0.32)	0.81 (0.22)	0.592 (2, 106)	0.555	6.455 (1, 53)	0.014*	0.341 (2, 106)	0.712
		RMS_CV.DIFF_ [1]	25	0.03 (0.18)	− 0.02 (0.16)	0.01 (0.19)	30	− 0.01 (0.14)	− 0.01 (0.09)	− 0.01 (0.08)	7.918 (2, 106)	0.001*	2.389 (1, 53)	0.128	0.537 (2, 106)	0.586
		RMS_CV.MEAN_ [1]	25	0.99 (0.46)	1.03 (0.45)	1.01 (0.38)	30	0.79 (0.25)	0.79 (0.36)	0.82 (0.23)	0.425 (2, 106)	0.655	7.797 (1, 53)	0.007*	0.178 (2, 106)	0.837

*Significant *p* value, *α* = 0.05. *N* number of subjects in statistical model, *IQR* interquartile range, *df*_*1*_ degrees of freedom for the number of comparisons within subjects, *df*_*2*_ degrees of freedom for the error term, *RMS*_*CV*_ relative cycle-to-cycle variability of muscle activity, *START* initial value, *DIFF* difference value between the start (rows 2 and 3) and end (rows 11 and 12) value

**Table 13:** Results of the post hoc within-subjects contrasts (day, day × sex) and between-subjects effects (sex) of the mixed analysis of variance (ANOVA) for absolute cycle-to-cycle variability of muscle activity with corresponding effect size *r* (Pearson’s correlation coefficient)

Parameter	Muscle	Outcome	Day									Sex			Interaction (day × sex)								
			Day 1 vs. 2			Day 1 vs. 3			Day 2 vs. 3			Men vs. women			Day 1 vs. 2			Day 1 vs. 3			Day 2 vs. 3		
			*F* value (df_1_, df_2_)	*p* value	*r*	*F* value (df_1_, df_2_)	*p* value	*R*	*F* value (df_1_, df_2_)	*p* value	*r*	*F* value (df_1_, df_2_)	*p* value	*r*	*F* value (df_1_, df_2_)	*p* value	*r*	*F* value (df_1_, df_2_)	*p* value	*r*	*F* value (df_1_, df_2_)	*p* value	*r*
RMS_CV_	M. extensor digitorum	START	1.77 (1, 54)	0.189	0.18	7.87 (1, 54)	0.007*	0.36†	4.41 (1, 54)	0.040*	0.27	14.75 (1, 54)	0.000*	0.46†	0.12 (1, 54)	0.729	0.05	0.10 (1, 54)	0.755	0.04	0.50 (1, 54)	0.481	0.10
		DIFF	50.64 (1, 54)	0.000*	0.70‡	23.46 (1, 54)	0.000*	0.55‡	0.77 (1, 54)	0.384	0.12	0.98 (1, 54)	0.325	0.13	0.00 (1, 54)	0.999	0.00	0.03 (1, 54)	0.859	0.02	0.05 (1, 54)	0.823	0.03
		MEan	3.31 (1, 54)	0.074	0.24	9.72 (1, 54)	0.003*	0.39†	2.28 (1, 54)	0.137	0.20	19.07 (1, 54)	0.000*	0.51‡	0.04 (1, 54)	0.837	0.03	0.23 (1, 54)	0.635	0.06	0.09 (1, 54)	0.770	0.04
	M. flexor carpi radialis	START	0.86 (1, 50)	0.358	0.13	3.30 (1, 50)	0.075	0.24	1.74 (1, 50)	0.194	0.18	2.70 (1, 50)	0.106	0.22	0.01 (1, 50)	0.903	0.02	0.01 (1, 50)	0.931	0.01	0.07 (1, 50)	0.786	0.04
		DIFF	118.41 (1, 50)	0.000*	0.83‡	0.94 (1, 50)	0.337	0.13	165.93 (1, 50)	0.000*	0.87‡	0.14 (1, 50)	0.709	0.05	0.01 (1, 50)	0.912	0.02	0.18 (1, 50)	0.669	0.06	0.10 (1, 50)	0.753	0.04
		Mean	0.00 (1, 50)	0.967	0.01	0.43 (1, 50)	0.515	0.09	0.78 (1, 50)	0.381	0.12	3.46 (1, 50)	0.069	0.25	0.48 (1, 50)	0.493	0.09	0.00 (1, 50)	0.948	0.01	0.61 (1, 50)	0.438	0.11
	M. biceps brachii	START	6.45 (1, 53)	0.014*	0.33†	5.28 (1, 53)	0.026*	0.30†	0.01 (1, 53)	0.904	0.02	7.65 (1, 53)	0.008*	0.36†	1.98 (1, 53)	0.165	0.19	0.17 (1, 53)	0.679	0.06	0.74 (1, 53)	0.394	0.12
		DIFF	5.63 (1, 53)	0.021*	0.31†	115.65 (1, 53)	0.000*	0.83‡	323.12 (1, 53)	0.000*	0.93‡	0.49 (1, 53)	0.486	0.10	0.07 (1, 53)	0.796	0.04	0.49 (1, 53)	0.486	0.10	0.35 (1, 53)	0.556	0.08
		Mean	1.35 (1, 53)	0.250	0.16	1.44 (1, 53)	0.235	0.16	0.26 (1, 53)	0.614	0.07	7.58 (1, 53)	0.008*	0.35†	0.71 (1, 53)	0.404	0.11	0.37 (1, 53)	0.543	0.08	1.79 (1, 53)	0.186	0.18
	M. triceps brachii	START	1.10 (1, 53)	0.300	0.14	0.32 (1, 53)	0.572	0.08	0.30 (1, 53)	0.589	0.07	6.45 (1, 53)	0.014*	0.33†	0.01 (1, 53)	0.924	0.01	0.43 (1, 53)	0.513	0.09	0.72 (1, 53)	0.401	0.12
		DIFF	1.63 (1, 53)	0.208	0.17	21.32 (1, 53)	0.000*	0.54‡	6.39 (1, 53)	0.014*	0.33†	2.39 (1, 53)	0.128	0.21	0.08 (1, 53)	0.776	0.04	1.42 (1, 53)	0.239	0.16	0.49 (1, 53)	0.485	0.10
		Mean	0.31 (1, 53)	0.579	0.08	0.66 (1, 53)	0.421	0.11	0.17 (1, 53)	0.683	0.06	7.80 (1, 53)	0.0007*	0.36†	0.27 (1, 53)	0.607	0.07	0.20 (1, 53)	0.656	0.06	0.00 (1, 53)	0.975	0.00

*Significant *p* value, *α* = 0.05. ^†^Medium effect size, *r* ≥ 0.3; ^‡^Large effect size, *r* ≥ 0.5. *df*_*1*_ degrees of freedom for the number of comparisons within subjects, *df*_*2*_ degrees of freedom for the error term, *RMS*_*CV*_ relative cycle-to-cycle variability of muscle activity, *START* initial value, *DIFF* difference value between the start (rows 2 and 3) and end (rows 11 and 12) value

For the FLEX, a main effect of day was found for RMS_CV.DIFF_ (*p* < 0.01; Table 12, Table 13, Fig. 5). The relative variability slightly increased within day 1, whereas it decreased within days 2 and 3 (*p* < 0.01). No main effect of sex was found for RMS_CV_ of the FLEX.

There were main effects of day for RMS_CV.START_ (*p* < 0.05) and RMS_CV.DIFF_ (*p* < 0.01) of the BIC (Table 12, Table 13, Fig. 5). The relative variability at start of the experimental task was higher on day 2 compared to day 1 (*p* < 0.05), and it decreased less within day 1 compared to days 2 and 3 (*p* < 0.01). A main effect of sex was found for RMS_CV.START_ (*p* < 0.01) and RMS_CV.MEAN_ (*p* < 0.01) of the BIC. Both RMS_CV.START_ and RMS_CV.MEAN_ were higher for men compared to women.

A main effect of day was found for RMS_CV.DIFF_ of the TRI (*p* < 0.01; Table 12, Table 13, Fig. 5). The relative variability increased within day 1 but decreased within days 2 (*p* > 0.05) and 3 (*p* < 0.01), and the decrease within day 3 was stronger than within day 2 (*p* < 0.05). There were main effects of sex for RMS_CV.START_ (*p* < 0.05) and RMS_CV.MEAN_ (*p* < 0.01) of the TRI. Females had lower RMS_CV.START_ and RMS_CV.MEAN_ than males.

No main day × sex interaction effects were found for RMS_CV_.

## Discussion

The aim of this study was to investigate whether aspects of motor control, i.e., neuromuscular responses and motor variability, during a non-fatiguing, repetitive screwing task, as reflected by muscle activity of various arm muscles, differed between females and males.

The first hypothesis of this study was that muscle activity would be higher and motor variability lower in women than in men, which was confirmed, since the static and median muscle activity levels of all muscles tended to be higher among women than among men. The results further supported our hypothesis that absolute motor variability of the flexor carpi radialis and biceps brachii and relative motor variability of the upper arm muscles were generally lower in women than in men. However, in contrast to our hypothesis, we found that the relative variability of the forearm muscles tended to be higher in women than in men. Our second hypothesis was that women would show less prominent changes in muscle activity and motor variability within and over the 3 days than men, which could not be confirmed by our findings. Instead, we found that upper arm median muscle activity levels tended to decrease within days among women but increase within days among men. Similarly, women showed a stronger decrease in absolute variability within days compared to men, who showed a weaker decrease or even increase within days.

### Methodological study aspects

When assessing the role of sex in the development of physical requirements and motor variability, it is very important that major confounders are ruled out. In our opinion, there are two such confounders. The first is *muscle strength*, which is known to be lower in women than in men [20]. To minimize the influence of muscle strength on our data, and therefore decrease the inter-subject variability due to muscle strength [29, 34], we have chosen to normalize to predefined reference force levels (i.e., RVCs) instead of to MVCs. To get an indication of the influence of normalization on the current dataset, we have post hoc calculated the average levels of RMS_10_, RMS_50_, and RMS_90_ expressed in both %RVE and %MVE. These values can be found in Additional files 1 and 2. The figures are intuitive: when normalizing to MVCs, the difference in average muscular load levels between men and women becomes extreme, which is mainly due to the differences in muscle strength between both sexes. For the simulated task in this study, women had to use more of their maximal muscle capacity to perform the screwing task than men for each of the four muscles (Additional file 2): triceps (4.18 vs. 1.09%MVE), flexor (15.99 vs. 7.19%MVE), biceps (21.50 vs. 9.99%MVE), and extensor (27.22 vs. 16.76%MVE). Similar findings of muscle activity normalized to MVC are reported by previous studies [6, 17]. The second confounder is the presence of *muscle fatigue* when performing a task. In being able to compare sex differences, it is very important to rule out any confounding effects of muscle fatigue. In some pilot measurements, the task was designed in such a way that we could be sure to avoid any development of muscle fatigue. However, we verified the non-fatiguing character of the repetitive task by showing that decreases of forearm muscle MVC and electromyographic manifestations of fatigue (i.e., concomitant increases in RMS with decreases in MF [31]) were both absent (see Table 3).

In this exploratory study, we have decided to use a mixed ANOVA for addressing potential differences between men and women with respect to different levels of muscle activity and motor variability. We have included an extensive set of outcome parameters that may be interrelated; however, we decided not to correct for this due to the exploratory approach of this study [35]. If future studies are assessing similar neuromuscular and motor variability aspects to investigate differences between men and women in light of their potential risk of developing MSD, the current findings need to be confirmed.

### Effect of sex on muscle activity

The static muscle activity level (RMS_10_) of forearm muscles during painting has been compared between men and women in a previous study [19], showing female painters requiring more static muscle activity than male painters. Note that Meyland et al. [19] normalized their EMG to the MVC. The median muscle activity levels (RMS_50_) of the flexor carpi radialis and triceps brachii were shown to be higher for women than for men, which confirms results of previous studies [18, 19]. These discrepancies between both sexes are present, despite EMG normalization to RVC instead of to MVC. Since especially a higher RMS_10_, and to a lesser extent a higher RMS_50_, has been related to a higher risk for developing MSD [36, 37], this may also apply to the results of the current study and contribute to the statistic that MSD are more prevalent among women than among men.

For the median muscle activity level (RMS_50_), an increase across an observation period can be interpreted in two ways. According to the one theory, it may be related to additional motor unit recruitment, changed motor unit discharge rates, decreased muscle fiber conduction velocities, and motor unit substitution [38, 39]. These characteristics may indicate the initiation of muscle fatigue, as supported by the results of two previous studies, in which a repetitive task until perceived fatigue (score of 8 on the CR10 Borg scale) was performed [40, 41]. According to the other theory, an increased RMS_50_ without a decreased MPF may point to a force increase [31]. In the current study, the RMS_50_ of the upper muscles tended to increase in men. Since we showed that muscle fatigue was absent (see Section 3.1), it is more likely that our male subjects tended to increase their force instead of initiating the process of muscle fatigue.

A decrease in RMS_50_ across an observation period may be related to a decreased central neural drive to the muscle [42], which is suggested to act as a protection mechanisms for the development of muscle fatigue [43, 44]. Decreased RMS_50_ has also been reported for forearm muscles [45] and for the upper arm and shoulder muscles [43, 46] along task performance. Although women showed higher RMS_10_ in several muscles than men in the current study, which is associated to a higher risk of developing MSD, they also showed tendencies for a decreased RMS_50_ along task performance within days, which may be seen as protection mechanism in developing muscle fatigue as potential precursor of MSD.

Nordander et al. [17] found peak muscle activity levels of the forearm muscles to be higher in females (39 %MVE) than in males (27 %MVE) when performing a full-day, heavy industrial task. This may be explained by the difference in muscle strength that is apparent between women and men. However, this explanation does not apply to the current findings that peak muscle activity of the triceps brachii was found to be higher among women (31.15 %RVE) than among men (25.64 %RVE), because muscle strength was excluded by an alternative normalization against an absolute reference voluntary contraction. The general activity level needed for the forward directed force during screwing, which is the main function of the triceps brachii, was very low (28.40 %RVE) and also much lower when compared to the other three arm muscles (extensor digitorum 71.22 %RVE; flexor carpi radialis 46.62 %RVE; biceps brachii 138.19 %RVE). These differences cannot be explained by factors such as working height or familiarization, since working height was individually adjusted to each subject’s elbow height and males and females were given the same time for task familiarization [23]. Therefore, one possible explanation could be that it has to do with socialization, whereby males are probably still more familiar with manual work than women [4]. This again may point towards both sexes applying different motor strategies when performing the same manual task [12], which is related to the margin of maneuver to perform the manual work in such a way that negative health consequences can be avoided or minimized [47].

In contrast, the RMS_90_ of the extensor digitorum and biceps brachii was higher in men than in women. Especially with respect to the biceps brachii being an important lower arm rotator, this may point to males focusing primarily on the more goal-directed, coordinating muscle in this screwing task. This has been previously suggested by others, based on the findings that muscle activity levels of assisting, secondary muscles during isometric contractions [48] and a box-folding task [12] were higher for females than for males and that muscle activity levels of the goal-directed, primary muscles during both tasks were higher for males than for females.

Initial and mean values for all muscle activity levels were highest on day 1 when compared to days 2 and 3. This finding is applicable to both men and women and may point toward motor skill learning, because the muscles may have learned to execute the same screwing task more efficiently [23, 49]. The only difference between sexes across days was found for the flexor’s initial peak muscle activity level (cf. Fig. 3), which was higher for men than for women on days 1 and 2, whereas it was higher for women than for men on day 3. This difference may point toward different motor skill development processes in men and women, with men being better able to improve the primary muscles involved in the task as has been previously suggested [48].

### Effect of sex on motor variability

It has been suggested that a lower motor variability may be associated with a higher risk for developing MSD [50]. The current results show that initial absolute variability of the extensor and biceps muscles was higher for men than for women. Similarly, the initial relative variability of the biceps and triceps was also higher for men than for women. The initial relative variability of the extensor, on the other hand, was higher for women than for men. Generally, men seem to have a higher variability at start of the screwing task, which would make them less prone to develop MSD while they might delay the fatiguing process in their muscles [41, 51, 52].

For the development of motor variability along the screwing task, this tends to mainly increase among men whereas it tends to decrease among women. This applies to both the relative as well as absolute motor variability. The motor variability patterns of the women in the current study are in contrast with those reported by Cid et al. [46] and Srinivasan et al. [22], who showed increased absolute and relative motor variability in both men and women. As muscle fatigue may influence the development of motor variability, this could be a factor explaining the discrepancy between the two studies [22, 46] and the current study. The differences found between men and women may actually point to both sexes applying different motor strategies [22].

Crucial to the course of motor variability is task duration and, in the long term, work experience. Previous studies have shown that the longer employees perform a job, the more variable their motor pattern tends to be [49, 53]. This aspect was covered in the current study by including 3 separate days of screwing for 1 h, with which we could display the initial development of motor variability. Our results indicate that absolute variability remained constant across the 3 days, whereas relative variability was generally higher on days 2 and/or 3 than on day 1. With respect to relative variability, these developments may imply that the participants learned to increase their motor flexibility in performing the screwing task [54]. However, with respect to absolute variability, it may also imply that the participants have been able to implement specific motor programs when performing the screwing task [53]. A third interpretation may include combining both variability and muscle activity level; a decreased muscle activity level with a stable absolute variability across days results in an increased relative variability and may point to economization of screwing performance. This was observed in the current study for the extensor muscle when comparing days 1 and 3 (cf. Table 4). These contrasting explanations clearly show that there is no consensus in the current literature whether either a decrease or an increase in motor variability should be considered as a risk factor for developing MSD [55, 56].

### Perspectives and significance

The simulation of repetitive screwing tasks has provided new insights into the level and development of muscle activity and motor variability in both men and women. However, when simulating work in the laboratory, motor control strategies that would be seen in real working environments may be influenced due to several organizational and psychosocial aspects of a real working environment being lost [12, 53]. In addition, the simulated 60-min screwing task did not reflect the job performed by, e.g., a carpenter or assembly worker, since these craftsmen probably will not screw 60 min in one piece, but may distribute it over the working day, depending on the work cycle or assignment. However, repetitive manual tasks in industry may require similar levels of muscular activation and cycle duration as the task studied in this study.

The prevalence of MSD tends to be higher in women than in men [1, 2]. Therefore, the risk factors for developing disorders have been explored to explain differences between men and women. The current study attempted to explain differences between both sexes using neuromuscular processes, i.e., muscle activity level and motor variability. However, other factors should also be considered, including other physiological reactions to repetitive work, organizational factors, social factors, and cultural factors [4]. When these factors can be evaluated simultaneously in a (simulated) work environment, this may provide a more complete picture of the nature of the differences between the sexes why women would be more susceptible to developing MSD then men.

This study is the first to compare differences between men and women in a relatively long-lasting simulated laboratory task on 3 different days. The advantage is that the levels of muscle activity and motor variability as well as the change along the 60-min task can be evaluated, as well as the change across days. With respect to changes across days, measurements were interspersed by 2 to 7 days. A minimum of 2 days was chosen, because it is known that performance improves across the following 24 h after practice [57] and across a good overnight sleep [58]. The inter-subject variation of the intervals between measuring days may have influenced the results, but we cannot determine to what extent.

## Conclusion

The current results showed that women generally have higher levels of static, median, and peak muscle activity than their male counterparts when performing the same repetitive, dynamic task. This implies that women may have a higher risk to develop MSD. In addition, the current results of both absolute and relative variability, although rather ambiguous, tend to show that women are more at a disadvantage with respect to the risk of developing MSD by showing lower initial motor variability than men. The intermuscular differences between men and women may point to both sexes having different intrinsic motor control strategies [5, 22, 48], emphasizing that biological aspects alone cannot explain why women would be at higher risk for developing MSD than men [59]. This means that a wider range of individual and environmental factors should be taken into account [4] as well as the full range of occupational tasks [56], so that work station design or work organization may be optimized not only at the sex level but also at the individual level.

## Supplementary information

**Additional file 1:** **Table S1.** The values for the different levels of muscle activity for men and women. IQR = interquartile range; RMS_10_ = 10^th^ percentile or static muscle activity; RMS_50_ = 50^th^ percentile or median muscle activity; RMS_90_ = 90^th^ percentile or peak muscle activity; RVE = reference voluntary electrical activity.

**Additional file 2: Figure S1.** Boxplots representing the 10^th^ percentile or static level (RMS_10_), 50^th^ percentile or median level (RMS_50_) and 90^th^ percentile or peak level (RMS_90_) of muscle activity for the biceps brachii, extensor digitorum, flexor carpi radialis, and triceps bracchii. Boxplots are shown for day 1 (white), day 2 (light grey) and day 3 (dark grey), for males and females, and for normalization to RVC (dashed) and MVC (solid).

## Data Availability

The datasets generated and analyzed during the current study are available in the tables as provided in this manuscript. Any additional information on the data can be requested from the corresponding author.
