# Supplementary material for: Sex differences in muscle activity and motor variability in response to a non-fatiguing repetitive screwing task
Source: Biol Sex Differ. 2020 Jan 28;11:6. doi: 10.1186/s13293-020-0282-2 (PMC6988371; doi:10.1186/s13293-020-0282-2)
Supplement: Supplementary file 1 — Additional file 1: Table S1. The values for the different levels of muscle activity for men and women. IQR = interquartile range; RMS10 = 10th percentile or static muscle activity; RMS50 = 50th percentile or median muscle activity; RMS90 = 90th percentile or peak muscle activity; RVE = reference voluntary electrical activity. [file 13293_2020_282_MOESM1_ESM.docx]

Table S1. The values for the different levels of muscle activity for men and women

|  |  | **Men** | | | | **Women** | | | |
| --- | --- | --- | --- | --- | --- | --- | --- | --- | --- |
|  |  |  | **Median (IQR)** | | |  | **Median (IQR)** | | |
| **Muscle** | **Outcome** | **N** | **Day 1** | **Day 2** | **Day 3** | **N** | **Day 1** | **Day 2** | **Day 3** |
| Biceps | RMS_10.MEAN_ [%RVE] | 26 | 1.74  (1.33) | 1.49  (1.42) | 1.72  (1.13) | 29 | 4.93  (3.57) | 4.63  (2.83) | 4.37  (3.76) |
|  | RMS_50.MEAN_ [%RVE] | 26 | 12.40  (10.23) | 9.69  (5.27) | 10.26  (7.24) | 29 | 24.76  (11.82) | 20.49  (14.51) | 20.01  (11.27) |
|  | RMS_90.MEAN_ [%RVE] | 26 | 51.64  (33.57) | 41.64  (27.15) | 42.00  (19.80) | 29 | 73.97  (30.56) | 65.29  (28.46) | 62.41  (20.05) |
| Extensor | RMS_10.MEAN_ [%RVE] | 27 | 5.90  (3.16) | 4.33  (2.48) | 3.95  (3.88) | 29 | 11.24  (5.52) | 8.31  (5.49) | 8.79  (4.23) |
|  | RMS_50.MEAN_ [%RVE] | 27 | 15.60  (10.07) | 17.11  (11.17) | 16.76  (12.52) | 29 | 28.99  (12.43) | 24.73  (13.26) | 24.61  (12.00) |
|  | RMS_90.MEAN_ [%RVE] | 27 | 28.02  (15.96) | 28.35  (19.19) | 28.93  (25.40) | 29 | 46.73  (20.19) | 41.73  (23.52) | 41.46  (20.56) |
| Flexor | RMS_10.MEAN_ [%RVE] | 27 | 2.08  (1.88) | 1.75  (1.46) | 1.04  (1.85) | 25 | 4.02  (2.89) | 4.16  (2.39) | 3.76  (3.36) |
|  | RMS_50.MEAN_ [%RVE] | 27 | 7.63  (5.74) | 6.74  (5.47) | 6.82  (5.73) | 25 | 16.69  (7.67) | 15.09  (12.00) | 13.94  (13.77) |
|  | RMS_90.MEAN_ [%RVE] | 27 | 18.82  (17.58) | 17.03  (16.37) | 16.87  (16.11) | 25 | 36.06  (24.68) | 36.86  (41.06) | 33.91  (29.92) |
| Triceps | RMS_10.MEAN_ [%RVE] | 25 | 0.51  (0.94) | 0.51  (0.70) | 0.52  (0.56) | 30 | 1.77  (1.24) | 1.69  (1.24) | 1.67  (1.10) |
|  | RMS_50.MEAN_ [%RVE] | 25 | 1.36  (1.77) | 1.04  (1.05) | 1.06  (0.82) | 30 | 5.05  (3.42) | 4.08  (4.05) | 3.58  (2.65) |
|  | RMS_90.MEAN_ [%RVE] | 25 | 6.76  (9.57) | 4.83  (6.55) | 4.39  (5.41) | 30 | 15.89  (14.90) | 14.46  (12.02) | 12.75  (9.69) |

*IQR* interquartile range; *RMS_10_* 10^th^ percentile or static muscle activity; *RMS_50_* 50^th^ percentile or median muscle activity; *RMS_90_* 90^th^ percentile or peak muscle activity; *RVE* reference voluntary electrical activity
