# Supplementary figures and images for: Sex differences in muscle activity and motor variability in response to a non-fatiguing repetitive screwing task
Source: Biol Sex Differ. 2020 Jan 28;11:6. doi: 10.1186/s13293-020-0282-2 (PMC6988371; doi:10.1186/s13293-020-0282-2)

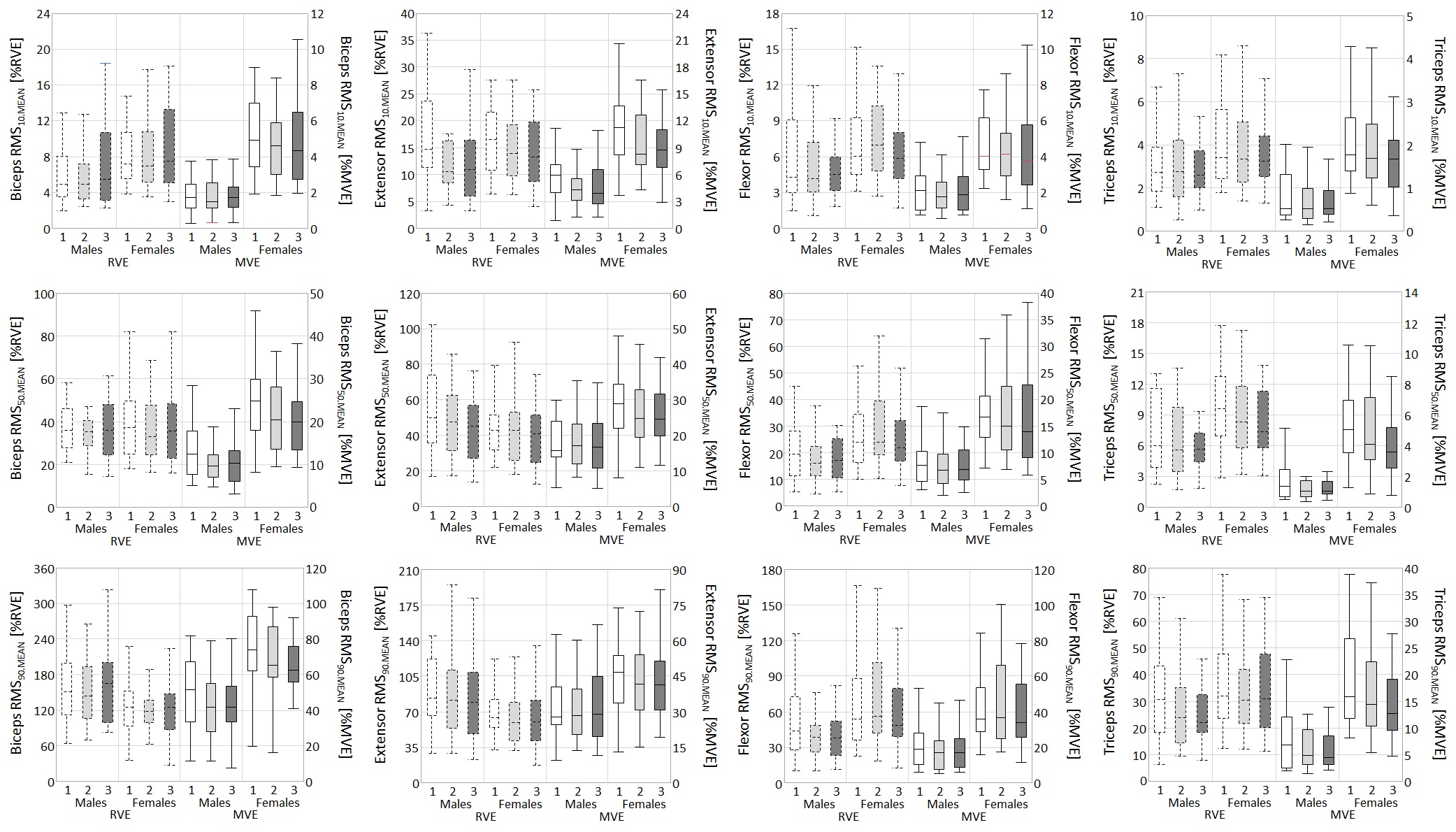

Supplement: Supplementary file 2 — Additional file 2: Figure S1. Boxplots representing the 10th percentile or static level (RMS10), 50th percentile or median level (RMS50) and 90th percentile or peak level (RMS90) of muscle activity for the biceps brachii, extensor digitorum, flexor carpi radialis, and triceps bracchii. Boxplots are shown for day 1 (white), day 2 (light grey) and day 3 (dark grey), for males and females, and for normalization to RVC (dashed) and MVC (solid). [file 13293_2020_282_MOESM2_ESM.jpg]
